# A Novel Approach to *Helicobacter pylori* Pan-Genome Analysis for Identification of Genomic Islands

**DOI:** 10.1371/journal.pone.0159419

**Published:** 2016-08-09

**Authors:** Ikuo Uchiyama, Jacob Albritton, Masaki Fukuyo, Kenji K. Kojima, Koji Yahara, Ichizo Kobayashi

**Affiliations:** 1 Laboratory of Genome Informatics, National Institute for Basic Biology, National Institutes of Natural Sciences, Okazaki, Aichi, Japan; 2 Department of Medical Genome Sciences, Graduate School of Frontier Sciences, University of Tokyo, Minato-ku, Tokyo, Japan; 3 Department of Computational Biology and Medical Sciences, Graduate School of Frontier Sciences, University of Tokyo, Minato-ku, Tokyo, Japan; 4 Institute of Medical Sciences, the University of Tokyo, Minato-ku, Tokyo, Japan; 5 Genetic Information Research Institute, Los Altos, California, United States of America; 6 Biostatistics Center, Kurume University, Kurume, Fukuoka, Japan; 7 Tohoku University, Graduate School of Life Sciences, Sendai, Japan; 8 Kyorin University, Faculty of Medicine, Mitaka, Japan; Institut National de la Recherche Agronomique, FRANCE

## Abstract

Genomes of a given bacterial species can show great variation in gene content and thus systematic analysis of the entire gene repertoire, termed the pan-genome, is important for understanding bacterial intra-species diversity, population genetics, and evolution. Here, we analyzed the pan-genome from 30 completely sequenced strains of the human gastric pathogen *Helicobacter pylori* belonging to various phylogeographic groups, focusing on 991 accessory (not fully conserved) orthologous groups (OGs). We developed a method to evaluate the mobility of genes within a genome, using the gene order in the syntenically conserved regions as a reference, and classified the 991 accessory OGs into five classes: Core, Stable, Intermediate, Mobile, and Unique. Phylogenetic networks based on the gene content of Core and Stable classes are highly congruent with that created from the concatenated alignment of fully conserved core genes, in contrast to those of Intermediate and Mobile classes, which show quite different topologies. By clustering the accessory OGs on the basis of phylogenetic pattern similarity and chromosomal proximity, we identified 60 co-occurring gene clusters (CGCs). In addition to known genomic islands, including *cag* pathogenicity island, bacteriophages, and integrating conjugative elements, we identified some novel ones. One island encodes TerY-phosphorylation triad, which includes the eukaryote-type protein kinase/phosphatase gene pair, and components of type VII secretion system. Another one contains a reverse-transcriptase homolog, which may be involved in the defense against phage infection through altruistic suicide. Many of the CGCs contained restriction-modification (RM) genes. Different RM systems sometimes occupied the same (orthologous) locus in the strains. We anticipate that our method will facilitate pan-genome studies in general and help identify novel genomic islands in various bacterial species.

## Introduction

Advances in DNA sequencing technology allow us to compare tens or even hundreds of genome sequences of related bacteria at once [1]. Such comparative genome analyses within a single bacterial species revealed substantial diversity in gene content, which posed the need for a concept of bacterial species genome [2]. To characterize genome diversity within a species, two terms have been commonly used: “pan-genome,” defined as the entire gene repertoire in a given species, and “core genome,” the set of genes conserved in all the strains [3]; the term “core genome” (or “core gene”) has also been used in a somewhat different, more relaxed sense (e.g., [4, 5], see below). According to this definition, the pan-genome consists of the core genome (in the strict sense) and the other part of genome called accessory (or dispensable) genome. Although the sizes of core genome and pan-genome have been successfully used as measures to evaluate intra-species diversity [6–8] and several tools have been developed for pan-genome analysis [9–11], these simple measures or tools alone are not sufficient to understand how each strain has evolved and how presence/absence of each gene contributes to the phenotypic differences between different strains. For these purposes, we need a more detailed and yet systematic approach to investigate the whole repertoire of pan-genome.

Synteny conservation provides useful information when comparing closely related genomes. A set of genes within syntenically conserved blocks in different strains can be considered another definition of “core genome,” in that they likely correspond to the genes conserved in the common ancestor that are inherited mainly through vertical transfer, while many of the genes acquired by horizontal gene transfer (HGT) are inserted into unpredictable positions. (In this consideration, we exclude inter-lineage transfer of an allele through homologous recombination from the definition of HGT). We previously developed a method (CoreAligner) to extract syntenically conserved regions and construct a core genome alignment between closely related genomes [5]. This information can be used as a basis for identifying vertically transferred genes. It is noteworthy that, in contrast to the above conventional definition of core genome as universally conserved (i.e., present in 100% of the strains) genes, the definition of syntenic core by CoreAligner allows inclusion of some accessory genes. This relaxation is needed to define core genome as covering a set of genes commonly found in typical strains. In fact, only 993 genes were conserved among all of the 61 sequenced *Escherichia coli* and *Shigella* spp. genomes [12], whereas a typical *E*. *coli* genome contains more than 4000 genes. Hereafter, we define the strictly conserved core as “universal core” to discriminate it from the “syntenic core” that includes accessory genes and use “core genome” mainly to designate the latter relaxed core.

On the basis of these terms, one can consider two approaches to assess intra-species diversity: one is to compare the sequences of the core genome and the other is to compare the contents of the non-core (accessory) genomes. The former approach is effective in inferring phylogenetic history because the core genome is inherited from the common ancestor and is conserved throughout evolution. In contrast, the latter approach, we focus on here, is more effective in understanding functional difference between strains that were generated through gain or loss of genes during evolution. Despite its importance in understanding intra-species diversity, characterization of non-core genes is relatively difficult because many of them are not assigned any function by homology-based analysis. Moreover, evolutionary relationships among non-core genes may be more complicated due to the occurrence of HGT.

Among the gene-content based comparison methods, phylogenetic pattern analysis (or phylogenetic profiling) is a “gene-centric” approach that classifies genes on the basis of their presence-absence pattern in different organisms [13]. It can predict functional relationships between genes and has been successfully used to infer hitherto unknown gene functions [14]. In the case of bacterial intra-species comparison, genes that have similar phylogenetic patterns, i.e. co-occurring exclusively in a particular set of strains, are often located close to each other and form a genomic island. Alternatively, gene content information can be used to calculate phylogenetic relationship among genomes [15, 16] although this approach can be misled by substantial gene loss and HGT [17]. To identify genes likely to have experienced HGT among different strains we introduced the simple concept of *mobility*. Mobility is defined here by the translocation of orthologous genes to different loci using the syntenic core as a reference coordinate.

In this work, we characterized the pan-genome of *Helicobacter pylori*, a human gastric pathogen that infects approximately half of the world population and causes several diseases such as gastritis, gastric/duodenal ulcer and gastric cancer [18]. *H*. *pylori* infection is usually chronic and mainly transmitted within families through oral ingestion in early childhood [18, 19]. *H*. *pylori* is known for high intra-species genetic diversity and rapid evolution through frequent mutual homologous recombination and high mutation rate [20]. *H*. *pylori* comparative genomics is particularly interesting because of its evolution through interaction with human hosts. They are likely to have evolved with *Homo sapiens* hosts through their migration out of Africa [21, 22]. On the basis of sequence comparison of several housekeeping genes, *H*. *pylori* strains were divided into populations associated with distinct phylogeographic groups such as hpEurope, hpEastAsia, hpAsia2, hpAfrica1 and hpAfrica2; hpEastAsia is further divided into subpopulations including hspEAsia, hspAmerind, and hspMaori. [21, 23, 24].

*H*. *pylori* is the first species for which complete genome sequences of two different strains were determined and compared [25, 26]. Previously, we compared complete genome sequences of 20 global *H*. *pylori* strains, including four newly sequenced Japanese strains, focusing on genomic features characteristic of the East Asian (hspEAsia) strains [27]. On the basis of phylogenetic pattern analysis and comparison of phylogenetic trees of individual core genes, we identified several genes whose differential presence/absence or sequence divergence patterns characterize East Asian strains. Although the phylogenetic tree constructed from the concatenated core genes shows strong congruence with the phylogeographic grouping previously identified, we realized that phylogenetic trees of individual core genes show deviation from the concatenated core gene tree [27, 28]. This is consistent with the panmictic, as opposed to clonal, nature of their evolution. Recently, we applied the *in silico* chromosome painting analysis to the genome-wide haplotype data generated from the alignments of the core genes among 29 global *H*. *pylori* strains to detect sequence sharing by homologous recombination and identify fine population structure [29].

Here, we explored the whole repertoire of *H*. *pylori* pan-genome, particularly the accessory genome. We focused on extracting sets of genes showing characteristic presence-absence patterns and compared them with phylogeographic grouping of *H*. *pylori* strains. In addition, we developed a novel method to identify mobile genes using the gene order in the syntenic core alignment as a reference coordinate. Combining these approaches, we identified and characterized several *H*. *pylori* genomic islands and discussed them in terms of their mobility.

## Materials and Methods

### Genome data and ortholog analysis

We used the complete genome sequences of 29 *H*. *pylori* strains reported in our previous study [29] plus the SouthAfrica7 strain [30]. The 29 strains were previously classified into 14 subgroups on the basis of the recombination analysis using the ChromoPainter program [31] (S1 Table). The SouthAfrica7 strain belongs to hpAfrica2 whose genome sequences are quite distinct from those of the other *H*. *pylori* strains. Orthologous groups (OGs) among the translated coding sequences identified in these genomes were generated using the DomClust program with the default parameter set [32]. The core genome alignment was generated using the CoreAligner program with the additional–SPCOV_SPRATIO = 0.2 parameter [5]. This parameter setting eliminates an alignment block that is completely lost in more than 20% of the genomes and is included in the current default parameter set in the MBGD database [33]. DomClust and CoreAligner were executed on the RECOG software (http://mbgd.genome.ad.jp/RECOG), an integrative environment for comparative genomics. This software allows us to manage the comparative genome database, execute DomClust and CoreAligner, perform clustering analyses (phylogenetic pattern clustering and neighboring gene clustering, see below) and visualize the results.

### Identifying co-occurring gene clusters

OGs were clustered on the basis of phylogenetic pattern similarity using a hierarchical clustering algorithm UPGMA implemented in RECOG (PhyloPatClust). Here, a phylogenetic pattern is represented as a binary vector consisting of 1’s (presence) and 0’s (absence). The dissimilarity *R* between two vectors is calculated as *R* = (1-*r*)/2, where *r* is the correlation coefficient of the two vectors (*R* ranges between 0 and 1). The clustering cut-off was set to *R*≤0.3, which is equivalent to *r≥*0.4.

To visualize chromosomal proximity of genes in the same cluster defined above, we used the “Neighboring Clusters” function in RECOG. For each genome (column), adjacent genes (within 10 rows) on the ortholog table (ordered here according to the output of the PhyloPatClust program) are assigned the same color, if they are closely located (within 5000 base pairs) on the chromosome (see Fig 5B–5F, below).

On the bases of these analyses, we created the *co-occurring gene cluster* (CGCs) as a set of OGs that belong to the same phylogenetic pattern cluster and are located in close proximity (i.e., belonging to the same neighboring cluster) in more than 50% of the strains. Here, we considered CGCs that have at least three OGs. The final set of CGCs was determined and annotated with manual curation using RECOG.

In addition, to further cluster CGCs that are closely located on the same chromosome, we created *neighboring co-occurring gene clusters* (NCGCs). This was done by merging a pair of CGCs if more than 50% of OGs in one of the CGCs are located close to the OGs in the other CGC. Two OGs are considered to be close when two genes, one from each OG, are located within 8000 base pairs on the same chromosome in more than 60% of the strains.

### Definition of mobility classes

A given OG is considered mobile when it can be found in multiple locations along a genome. To evaluate OG mobility, we developed the FindMobile program that uses a core genome alignment generated by CoreAligner [5] to define a reference coordinate and compares the positions of genes for each non-core OG using this reference coordinate.

**Fig 1:**
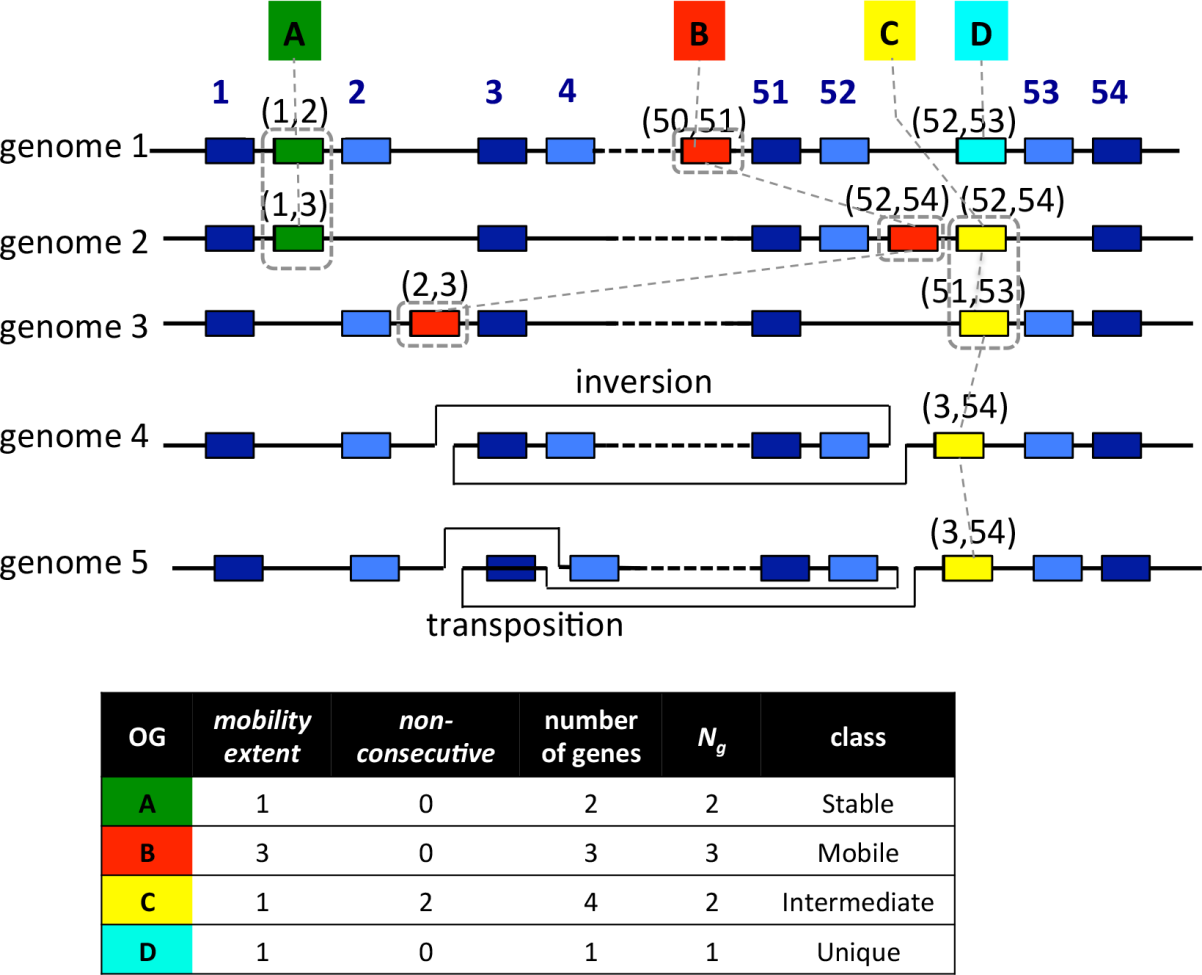
Definition of the mobility classes. Genes in the (syntenic) core OGs include the universal core genes (boxes in dark blue) and the remaining syntenic core genes (boxes in pale blue). Each core OG is assigned a core index (the above number) representing its order in the core alignment. Each of the non-core OGs (A, B, C, and D boxes) is assigned a pair of core indices representing the left- and right-neighboring core OGs. A set of genes that are located in the equivalent locus is enclosed in a box of a dashed line. The *mobility_extent* of each OG is defined as the number of distinct loci where the OG can be located, which is one for OG-A, three for OG-B, and one for OG-C. Note that we ignored the genes in OG-C in genomes 4 and 5 in which the difference between the left- and right-neighboring core indices is too large (*non-consecutive*), which indicates that the gene is located around a break point of a large rearrangement (in these cases, inversion and transposition). OG-D appears in only one genome and thus is classified as Unique. Mobility class is defined on the basis of *mobility_extent* and *N*_*g*_ (see text), where *N*_*g*_ is the effective number of genes obtained by the following calculation: (number of genes in OG)–*non-consecutive*.

A core genome alignment represents the consensus order of OGs that are conserved in at least 50% of the genomes, and its order is determined on the basis of the neighboring OG pairs in which genes are closely located in at least 50% of the genomes [5]. Each core OG is assigned an index according to its position in the core alignment, and each gene belonging to the *i-th* core OG is assigned the position *i* (Fig 1). Sometimes there are duplicates within a genome (inparalog) in a given OG. In such a case, FindMobile tries to pick a gene in the orthologous position by checking whether the nearest core genes on either side of the chromosome form an ascending or descending sequence of core indices within a cut-off distance.

Next, for each gene in the OGs that are not included in the core alignment and are not unique (we classify OGs present in only one strain in a distinct class as Unique; see Fig 1), we assigned a relative core position. For this purpose, the nearest core genes of the target gene on either side of the chromosome are identified and the core indices of these neighboring genes is recorded as an interval denoted as (*i*, *j*) where *i* < *j* (see non-core, non-unique OGs A-C in Fig 1). Then, mobility of a given non-core OG is evaluated by comparing the relative core positions among the genes in the OG. Since CoreAligner allows some exceptions in the conservation of gene order during the construction of a core alignment, the resulting core alignment can contain a rearrangement. If the two indices (*i*, *j*) of the neighboring core genes are far apart from each other, i.e., |*j*-*i*| > *c* with a given cut-off *c* (condition *non_consecutive*), there is likely to be a rearrangement point between these core genes (see OG-C in genomes 4 and 5 in Fig 1). Therefore, FindMobile excludes such a case from this evaluation.

In addition, we define *compatibility* of intervals as follows: two intervals *r*_1_ = (*i*, *j*) and *r*_2_ = (*k*, *l*) are compatible if they have some overlap, i.e., *i* < *l* and *k* < *j*. In such a case, the difference in the intervals on which orthologous genes are located can be explained only by small deletions and/or insertions. In Fig 1, the two genes in OG A are compatible whereas the three genes in OG B are not compatible with each other. In the latter case, a translocation is required to explain the observed position difference of the target genes. We applied a single linkage-clustering algorithm to group genes in compatible locations and defined *mobility_extent* of OG as the number of distinct locations obtained by this analysis. A gene located on a plasmid that has no core gene cannot be assigned a location, but is considered in a distinct location, and thus it also increments *mobility_extent*.

Finally, FindMobile classifies non-core accessory OGs into three categories: Stable, Mobile, and Intermediate. We classified an OG into Stable class when *mobility_extent* = 1 and no gene is located in an interval satisfying the *non_consecutive* condition. Thus, the positions of all the genes in a Stable OG are fixed in the core alignment and are not affected by transpositions or rearrangements. The remaining OGs are classified into either Mobile (highly mobile) or Intermediate (occasionally mobile or questionable) according to the value of *mobility_extent*. Since sometimes an apparent gene transposition may occur through repeated genome rearrangements, such as inversions, we intend to discriminate such cases from transpositions through mobile genetic elements where *mobility_extent* should be much higher than the former cases. We classified OGs into Mobile class if *mobility_extent* = 2 and *mobility_extent* > 0.5 *N*_*g*_; *mobility_extent* = 3 or 4 and *mobility_extent* > 0.3 *N*_*g*_; or *mobility_extent* > 4, where *N*_*g*_ is the number of genes in the OG that do not satisfy the *non_consecutive* condition. Otherwise, we classified them into Intermediate class.

### Phylogenetic analysis

To conduct phylogenetic analysis of core genes, we consider a set of core OGs that are conserved in all strains, in one-to-one relationship, and contain no domain splitting. A multiple alignment was calculated for each core OG using the MAFFT program with default parameters [34]. Concatenated core alignments were then constructed after eliminating gapped sites. Phylogenetic networks were calculated using the NeighborNet method [35] implemented in the SplitsTree program [36].

For phylogenetic analysis based on gene content, the presence/absence of each OG in each strain is represented by a binary vector where presence and absence are coded as 1 and 0, respectively. The resulting character matrix is used as an input of the SplitsTree program to construct a phylogenetic network.

## Results

### The pan-genome and core genome identified among 30 *H*. *pylori* strains

**Fig 2:**
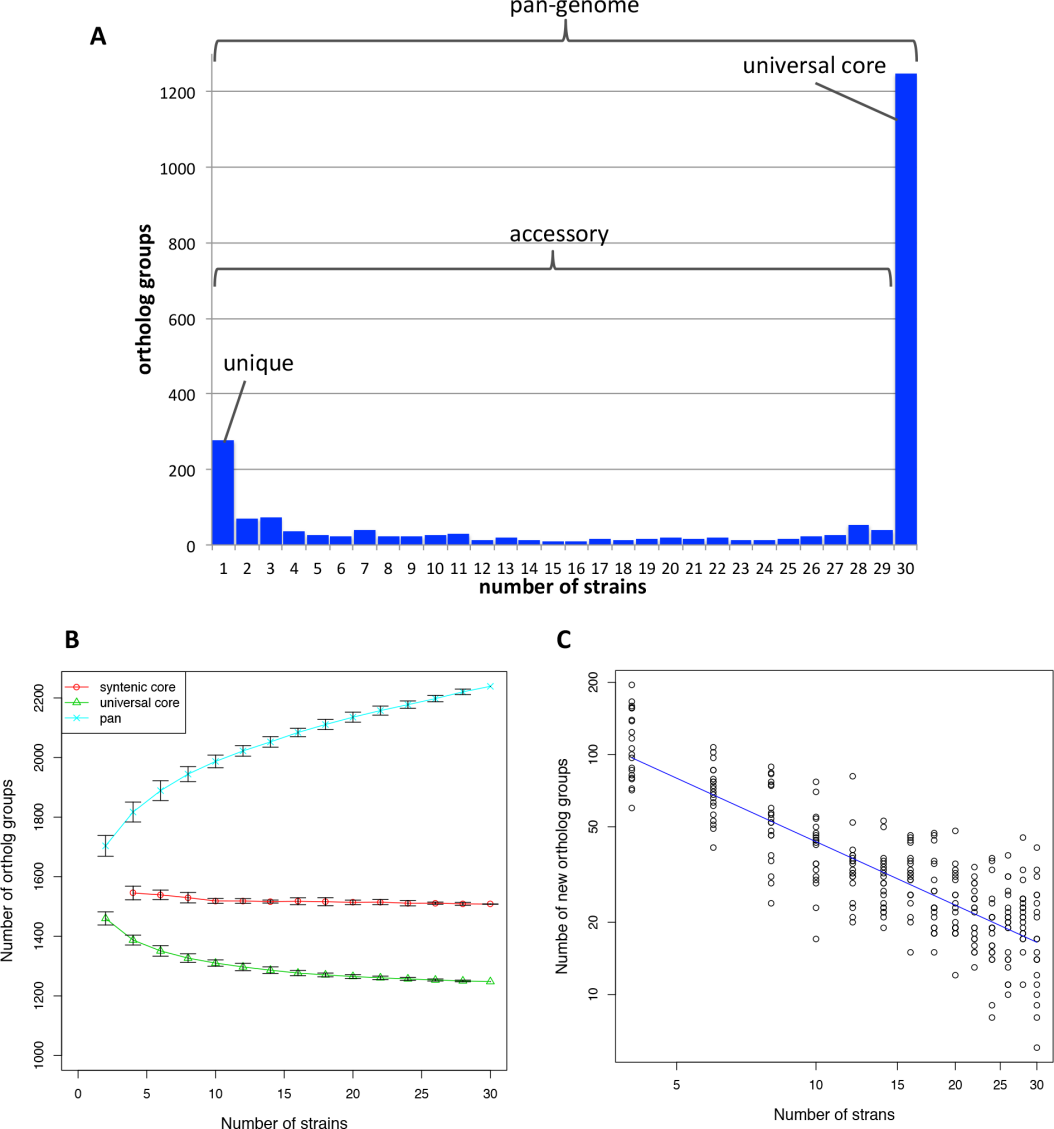
Pan-genome and core genome among *Helicobacter pylori*. (A) Histogram showing the distribution of the number of strains in each OG among the 30 strains. Sets of OGs corresponding to pan-genome, universal core, accessory, and unique OGs are indicated. (B) Sizes of the syntenic core, universal core, and pan-genome as functions of the number of strains. An ordered lists of the 30 strains was randomly generated and the sets of *n* strains (*n* = 2,4,…,30) generated from this list was subject to core- and pan-genome analysis. The test was repeated 20 times and the average numbers of core- and pan-genome sizes were plotted with error bars that represent standard deviations. Syntenic core between two genomes is not well defined and thus is not plotted. (C) The number of new OGs added to the pan-genome as a function of the number of strains. The number of new OG in *n* strains (*n* = 4,6,…,30) was calculated as the difference between the pan-genome size in *n* strains and that in *n–* 2 strains.

Orthologous clustering of the genomes of 30 *H*. *pylori* strains identified 2239 OGs defining the pan-genome. Among them, 1248 OGs are conserved in all the 30 strains, and represent the universal core. The remaining 991 OGs correspond to the accessory genome in which 277 OGs are unique (i.e., OGs present in only one strain) (Fig 2A).

In addition, the syntenic core was constructed using the CoreAligner program [5] by setting the conservation cut-off to 0.5 (default setting). Thus, the syntenic core is constructed using the OGs that are conserved in at least half of the genomes and the OG pairs that are closely located (within 20 genes) in at least half of the genomes. Because of this relaxed conservation condition, the number of genes in the syntenic core is generally larger than that of the universal core, provided that the synteny is well conserved. Here, the size of the syntenic core among the *H*. *pylori* strains is 1499 OGs including 254 accessory genes. Three universal core OGs were excluded from the syntenic core due to the existence of many inparalogs that obscures their positions in the alignment.

Fig 2B is a plot showing the sizes of pan-genome, universal core, and syntenic core as functions of the number of genomes that were added in a random order. When taking the increasing rate from 26 to 30 strains, the size of the syntenic core is almost constant, whereas the size of the universal core is decreasing slowly. On the other hand, the pan-genome size is increasing more definitely at the rate of approximately 20 OGs/genome. By fitting the power law distribution (*Δn ~ N*^*–α*^) to the relation between the increase in the number of OGs (*Δn*) and the number of genomes (*N*), we obtained the coefficient *α* = 0.879±0.035 (Fig 2C). The coefficient *α*≤1 indicates that the pan-genome of *H*. *pylori* is “open” i.e., the size of the pan-genome tends to diverge when *N* increases [37], as concluded in a previous analysis using seven *H*. *pylori* genomes [38].

### Mobility of genes based on relative positions in the core genome alignment

To characterize the accessory OGs in the *H*. *pylori* pan-genome, we consider the “mobility” of genes (OGs), which is defined here as whether genes in a given OG appear to have transposed on the genome. We can infer that such genes can move within or between genomes during evolution although we cannot detect a mobile element with high insertion site specificity by this definition. For this purpose, we developed a simple program (FindMobile) that evaluates the mobility of each OG by comparing the locations of its orthologs in different genomes on a common reference coordinate based on the core genome alignment (Fig 1). In this evaluation, all genes included in the core alignment are assumed not to be mobile and are classified into “Core” class. We also classified unique OGs (present in only one strain) into a distinct “Unique” class. For each of the other accessory OGs, FindMobile records the positions (the order in the core alignment) of the neighboring syntenic core genes on both sides on the chromosome and compares the positions of genes within the same OG. On the basis of this comparison, FindMobile classifies each non-Core and non-Unique accessory OG into one of the three classes: Mobile, Stable, and Intermediate (Fig 1). In a Stable class OG, all the genes are located in the same locus (defined as a position on the reference coordinate), whereas in a Mobile class OG, genes are located in several distinct loci, so that they are likely to be part of a mobile element. Intermediate class includes questionable cases due to genome rearrangements (see below) and other events. Here the number of distinct loci on the reference coordinate for a given OG is defined as *mobility_extent*, and we classified OGs into Mobile class when their *mobility_extent* is high enough (see Materials and Methods). Remaining OGs are classified into Intermediate class. Sometimes the adjacent core genes on both sides of a target gene are not consecutive in the core alignment. This indicates that genomic rearrangement occurred around the target gene and, in such a case, we cannot locate the gene on the reference coordinate (genome 4 and 5 in Fig 1). Consequently, an OG containing genes located in regions flanking a rearrangement boundary is classified into Intermediate class as a questionable case even if the remaining genes are located in the same locus. On the other hand, an apparent transposition may also result from repeated inversions rather than the mobility of the genomic island itself. To eliminate such possibility, we also classify an OG of low *mobility_extent* into Intermediate class as a questionable case.

**Fig 3:**
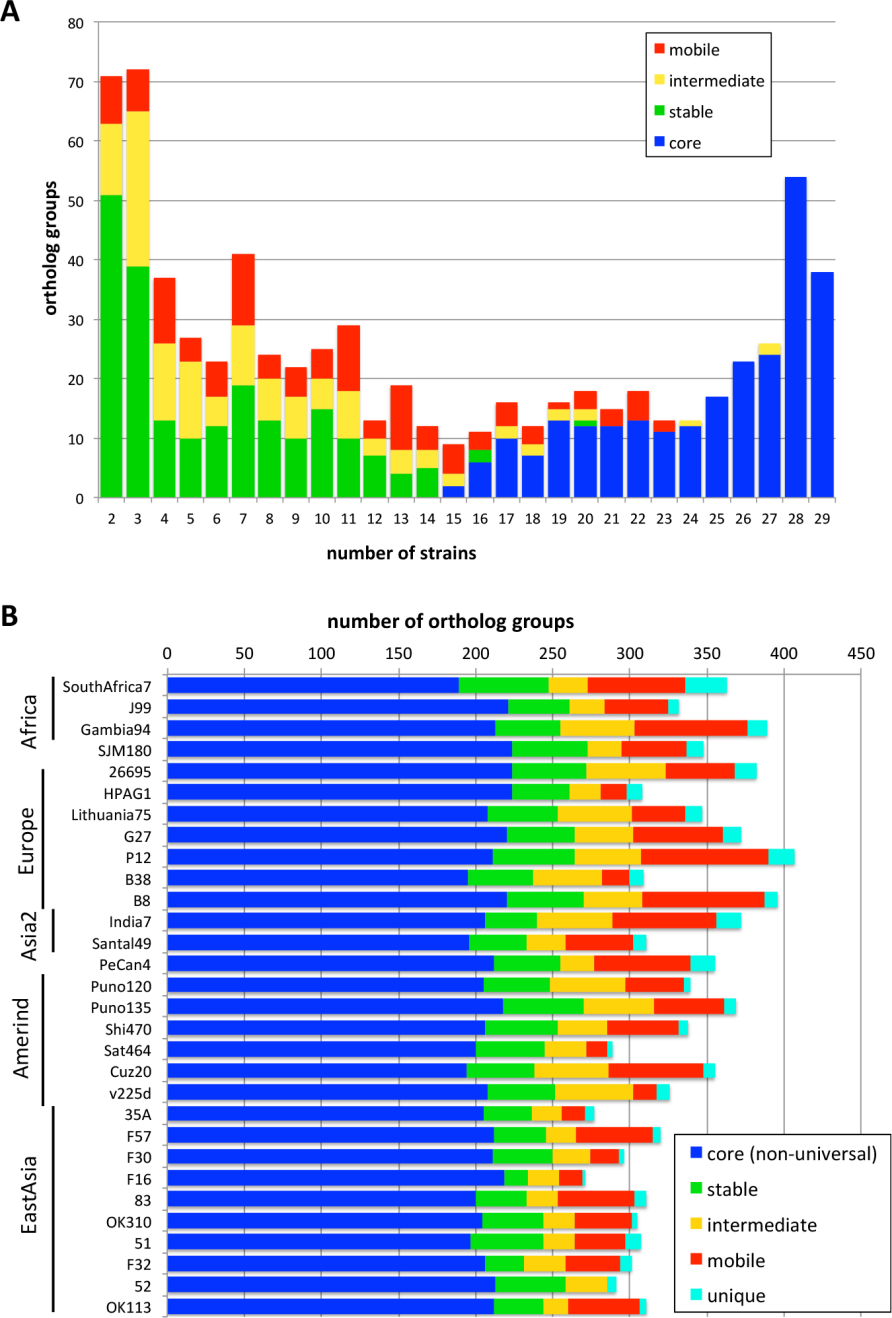
The number of OGs classified in each mobility class. (A) Histogram showing the strain number distribution of each mobility class among non-unique accessory OGs. The histogram is equivalent to Fig 1(A) except the rightmost bar representing the universal core OGs (*num_strain* = 30) and the leftmost bar representing the unique OGs (*num_strain* = 1) are eliminated. (B) Frequencies of the mobility classes among the accessory OGs in each strain. The order of strains is same as in S1 Table. Note that each strain also has the same number (1248) of universal core OGs that are not shown in this graph.

We classified 991 accessory OGs into these mobility classes and obtained 254 Core, 211 Stable, 129 Intermediate, 120 Mobile, and 277 Unique class OGs (S2 Table). Fig 3A illustrates the distribution of the number of strains for each mobility class assigned to the non-unique accessory OGs. Since the conservation cutoff in the CoreAligner program is 50%, most of the conserved OGs (i.e., OGs containing more than or equal to 15 strains) were classified in the Core class, although some conserved OGs were classified in different classes (mostly Mobile class). Note that the OGs conserved in more than half of the strains but not contained in Core (defined by the CoreAligner program) can be classified in the Stable class by our definition. On the other hand, non-conserved OGs (i.e., OGs containing less than 15 strains) are not included in Core by definition, but many of them were classified in Stable class because they were located in the equivalent positions of the core alignment. Stable class constitutes approximately 50% of the non-conserved OGs, while the remaining OGs are either Mobile or Intermediate class.

The actual numbers of accessory genes in each class are different among strains (Fig 3B). This seems to be due to the highly variable numbers of Mobile genes that ranges from a maximum of 81 in strain P12 to zero in P52. In addition, the number of Intermediate genes is also variable and its small size appears to be the main cause of the smaller number of accessory genes in East Asian strains. In contrast, the numbers of Core and Stable genes are relatively constant among strains.

### Phylogenetic network based on gene content

**Fig 4:**
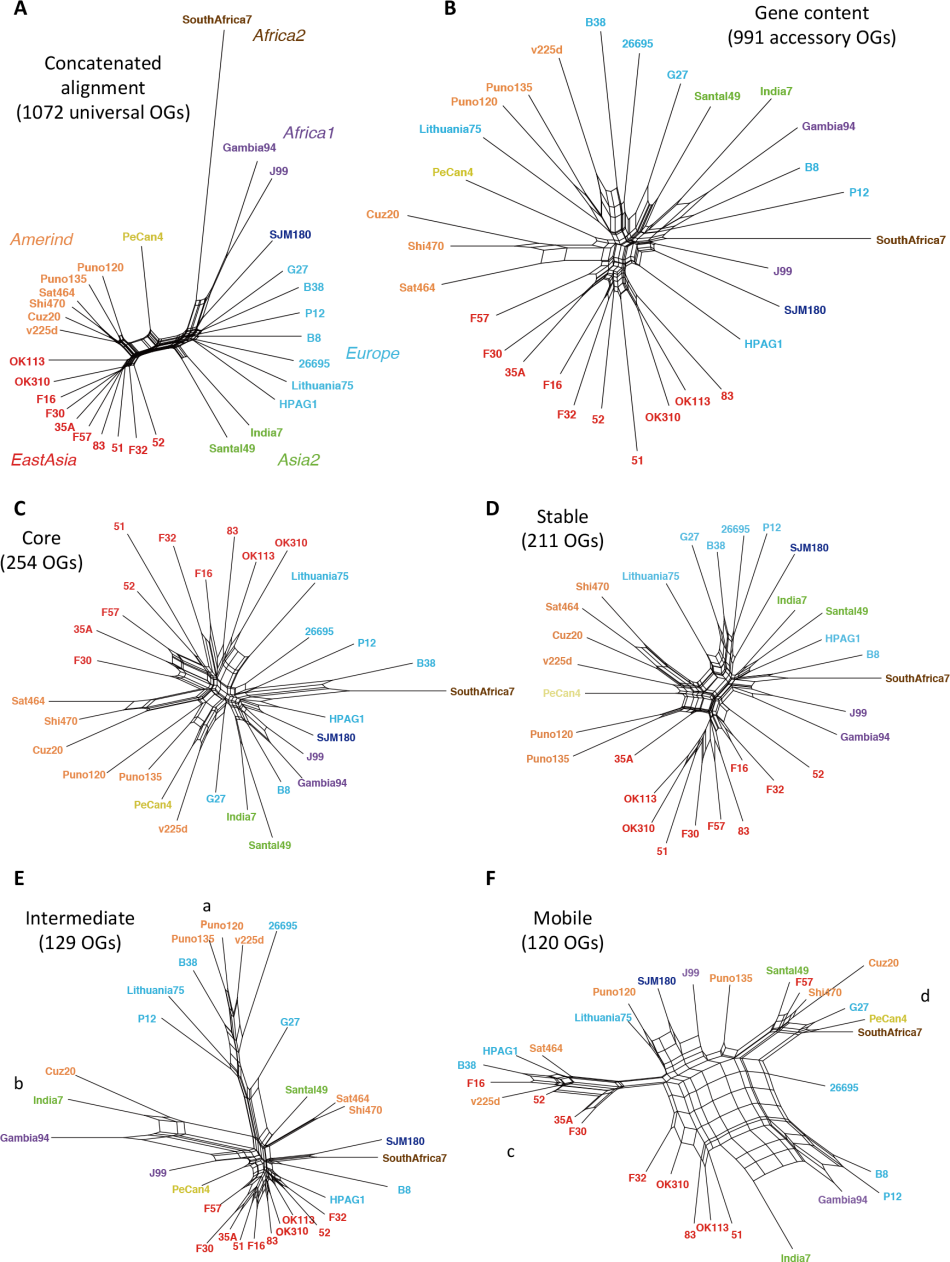
Phylogenetic networks among 30 *H*. *pylori* strains. (A) From the concatenated alignment of the universal core OGs. (B) From the gene content (presence vs. absence) of the entire accessory OGs. (C) From the gene content of Core class OGs. (D) From the gene content of Stable class OGs. (E) From the gene content of Intermediate class OGs. (F) From the gene content of Mobile class OGs. Strain names are assigned colors according to the phylogeographic groups as follows: brown, Africa2; purple, Africa1; dark blue, SJM180; light blue, Europe; green, Asia2; khaki, PeCan4; orange, Amerind; red, East Asia.

Accessory gene content information can also be used to construct a phylogenetic tree [15] and this approach is applicable to comparison of closely related genomes [39]. Here, we consider phylogenetic networks rather than trees to visualize non-tree like relationships that include HGT. We created a phylogenetic network using the NeighborNet method [35] with a character matrix representing the presence/absence of accessory OGs in each genome (Fig 4B) and compared it with the phylogenetic network created from the concatenated alignment of the universally conserved core OGs (Fig 4A), which is a more conventional approach to analyze phylogenetic relationships using the whole genome information. As previously shown [29], the (Fig 4). The geographic groups and subgroups are well separated on the phylogenetic network constructed from the concatenated core alignment, as previously shown [29]. Despite completely different sources of information used to construct the network, we were also able to identify some clusters belonging to one particular geographic group on the gene-content-based network (Fig 4B). However, separation among the groups is not perfect, except in the East Asian group, which forms a distinct cluster on the network (Fig 4B). A plausible reason for this difference is the higher susceptibility to the effect of HGTs of the gene content method using non-core genes, compared with that of the concatenated core alignment method.

To demonstrate the effects of HGTs on group clustering, we created a phylogenetic network using the gene content matrix for each of the above-defined four classes separately: Core, Stable, Intermediate, and Mobile (Fig 4C–4F). As expected, the geographic groups are better clustered in the networks of Core and Stable classes than those of the other classes. In fact, in the networks of Core and Stable classes, not only the East Asian group but also the Amerind group forms distinct clusters (Fig 4C and 4D). Note that PeCan4 is a hybrid between the European and Amerind strains [29].

In the phylogenetic network of the Intermediate class, while East Asian strains form a compact cluster, there are two distinct clusters that consist of strains in different geographic groups: one comprising three Amerind strains and five European strains (indicated by a in Fig 4E) and the other comprising three strains from Amerind, Asia2, and Africa1 (indicated by b in Fig 4E). In the phylogenetic network of Mobile class, clusters of geographic groups as well as tree-like structure are further lost and the central reticulated structure occupies a large space (Fig 4F), suggesting the dominance of horizontal rather than vertical transfers in the Mobile class OGs as expected.

### Co-occurring gene clusters (CGCs) based both on similarity of phylogenetic patterns and chromosomal proximity

**Fig 5:**
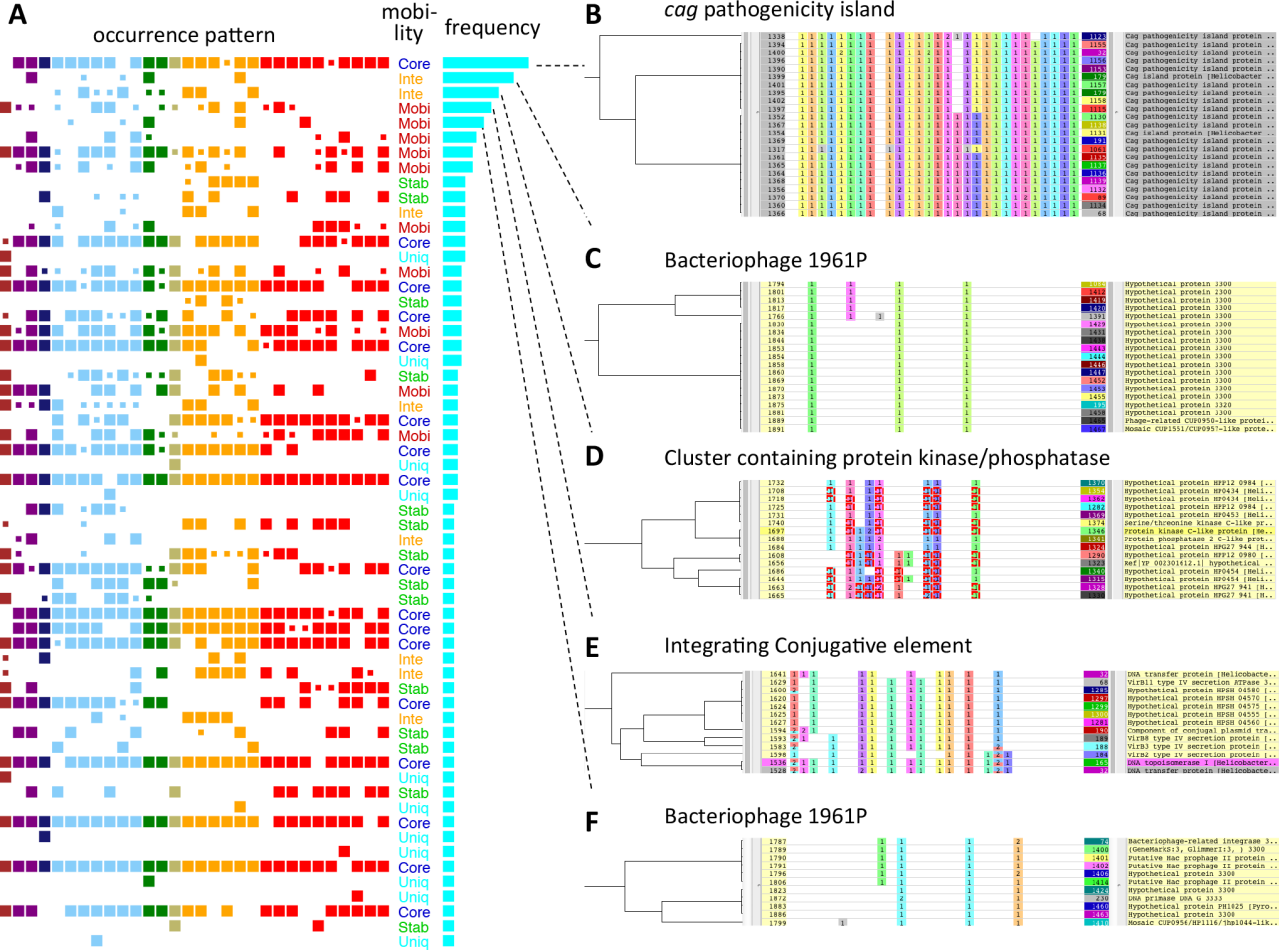
Co-occurring gene clusters (CGCs). (A) The 60 CGCs ordered according to the cluster size (the number of OGs included). An occurrence pattern represents presence/absence of CGC in each strain where a large box indicates that the strain contains all OGs in the CGC and a small box indicates that the strain contains only part of the OGs. In the occurrence pattern, strains are ordered in the same way as in S1 Table and colors are assigned according to the phylogeographical groups in the same way as in Fig 4. (B-F) The five largest CGCs displayed on the RECOG system. (B) CGC-1 corresponding to *cag* pathogenicity island; (C) CGC-2 corresponding to a part of bacteriophage 1961P; (D) CGC-3 containing protein kinase C and protein phosphatase C2 homologs; (E) CGC-4 corresponding to a part of ICE containing type IV secretion system; (F) CGC-5 corresponding to a part of bacteriophage 1961P. The left part shows a hierarchical clustering tree based on the occurrence pattern similarity. The central part shows occurrence patterns, where the order of strains is same as in (A), and the colors are assigned according to the neighboring clustering, i.e., the cells filled in the same color in each column contain genes that are closely located on the chromosome (here, we used 8000 bp window for the neighborhood criterion). Enlarged figures B-F are shown in S1 Fig.

We compared the phylogenetic patterns of accessory OGs to classify them. There were 527 unique phylogenetic patterns among 714 non-unique accessory OGs. To further classify the patterns, we performed hierarchical clustering based on the correlation coefficient between a pair of phylogenetic patterns. The resulting clusters were analyzed by the Neighboring Cluster function in the RECOG program and further divided such that each cluster corresponds to a distinct neighboring cluster (see Materials and Methods and Fig 5B–5F). Thus, we obtained 60 CGCs that consisted of at least three OGs each and 303 OGs in total (Fig 5A, Table 1 and S2 Table). In addition, we defined NCGCs by merging CGCs that are closely located on the same chromosome and obtained 8 NCGCs that consisted of at least two CGCs each (Table 1 and Table 2).

**Table 1:** Co-occurring gene clusters (CGCs).

CGCID	NCGCID	NumOGs	Occurrence pattern^a^	Comments	Mobility^b^	RM^c^
1		23	_BBCDDDDD_DEEFGGGgGGHHHHHhHHHH	cag pathogenicity island	core[23]	
2	2	19	__B___.__._E______G___________	Bacteriophage 1961P	intermediate[18], mobile[1]	
3	3	15	____d_DddD_.._GG___G__________	Amerind+Europe; TerY-P triad cluster incl. Ser/Thr protein kinase and protein phosphatase	intermediate[15]	IV
4	1	13	A.b_.__DD_d_ef_gG_G_.H._______	ICE; type IV secretion system tfs4	mobile[13]	
5	2	11	_____.___d_E______G____H______	Bacteriophage 1961P	mobile[10], intermediate[1]	
6	1	9	__B_____D_De____________h_H__h	ICE; type IV secretion system tfs3	mobile[9]	
7	1	8	ABBCd_DDD_DEEfGgG.G__H__hHhH_H	ICE; type IV secretion system (common in tfs3 and tfs4)	mobile[8]	
8	1	8	_bBC._D_d_DE__gG_.______hH_H_H	ICE; relaxase, protease, gyrase	mobile[8]	
9		6	______________._GGGG__________	Amerind specific; incl. Exodeoxyribonuclease VII large subunit and HNH/ENDO VII nuclease	stable[5], intermediate[1]	
10		6	___C___._..___G_G_____H__HH__H	incl. reverse transcriptase and phage-associated protein	stable[6]	
11	3	6	____D____d____GG___G__________	Amerind x 3, Europe x 2; incl. AAA family ATPase	intermediate[6]	
12	1	6	__b_d___D_dE____________HHHh_h	ICE; type IV secretion system tfs3; VirB2, VirB3, VirB4	mobile[6]	
13		6	aBBCD_DDDDDEEF_GGGGG___HHHhHHH	DNA exonuclease RecJ, conserved domain DUF262	core[6]	
14	1	6	A_____________________________	SAfrica7 specific; incl. type IV secretion system protein VirB11	unique[6]	
15	1	5	A_B.d_.DD_dEeF_gG_G__H__._H__.	ICE; relaxase VirD2, conjugal transfer protein TraG, VirD4	mobile[5]	
16	6	5	ABBCDDdDDDDEeFGGGGGGHHHHH__HHH	Hypothetical (putative ATP-ase or ATP/GTP-binding protein)	core[5]	
17	4	5	______________gG_Gg___________	N-acetylneuraminic acid synthetase, N-acylneuraminate cytidylyltransferase, sialyltransferase	stable[5]	
18		5	a_BC_DDDd.D.E_G__G_g__HHHH_H_H	Dam and other restriction endonuclease and methyltransferase	core[5]	II
19	1	5	AbBCD_DDD_DEeFGGG_G_HHH_hH_h_h	ICE; DNA topoisomerase, Integrase/recombinase, toprim-like family protein	mobile[5]	
20	5	5	ABBCDDDDDDDEEFGG___GhHHHHH_HHH	DnaK homolog, WxG100 family	core[5]	
21		5	_______________G______________	Puno135 specific, urease alpha/beta, phage resistance protein RloAB	unique[5]	
22		4	A____dDDdDdeeF__GGgg________H_	Hypothetical	stable[4]	
23	1	4	ABBC___DD_D_EF__G_G__H__H_____	ICE; VirB6	mobile[4]	
24	3	4	A..CD_. . .dd___GG___G__________	AAA ATPase	intermediate[3], mobile[1]	
25		4	____D_DdDD___FGGGGggHHHHHHHhHH	Phage lysozyme	core[4]	
26	1	4	A_B_D__DD_DEef__g_g_.Hh_HHHH_H	ICE; VirB4-2	mobile[4]	
27	6	4	ABBCDD.DDDDE.FGGGGGGH_H_______	incl. CrfC homolog (dynamin-like GTPase family)	core[4]	
28	7	4	_____________F________________	PeCan4 Specific, methyltransferase, Type II restriction endonuclease	unique[4]	II,III
29	4	4	ABBCDDDD_DDEEFGGGGGGHHHHHHHHHH	Thiamine biosynthesis, hsdR	core[4]	I
30		4	________D_____________________	P12 specifc; Chorismate synthase,pyrophosphatase,menaquinone biosynthesis protein	unique[4]	
31		3	__BCD_.DD.____________________	Type II restriction endonuclease and methyltransferase	stable[3]	II
32		3	a_____._______GG__G_H_H_HHH___	Site-specific DNA methylase Dcm	stable[2], intermediate[1]	II
33	1	3	_bB_____D_D___________________	Hypothetical (incl. weak homolog of tyrosine recombinase XerC)	intermediate[3]	
34		3	Ab____d______FGGgggGhHH_______	Hypothetical	stable[3]	
35	5	3	ABbCDDDDDDDe.FGG___G___HHhH_HH	Hypothetical (incl. weak homolog of chromosome segregation protein SMC)	core[3]	
36	7	3	____D__DDD_EEf_____G__________	Type III restriction endonuclease and methyltransferase	stable[3]	III
37		3	A__cDD__DDd.__________________	Hypothetical (OMP)	stable[3]	
38		3	_BBCDDDDDDDEEFGGGGGGHHHHHhHH_H	Type II methyltransferase	core[3]	II
39		3	_BBCDDDD__DEEFGGGGGGHHhhHHH_HH	Type II restriction endonuclease and methyltransferase	core[3]	II
40		3	ABBCdDDDDDDEEF_G_GGGHHHHHHH_HH	Hypothetical	core[3]	
41		3	.__C____________G_G___________	Hypothetical	intermediate[3]	
42	4	3	._________D_E__GGGG_H_H____Hh_	Type II restriction endonucleas and methyltransferase	intermediate[2], stable[1]	II
43		3	A_B_______D____________HhhHHHH	Predicted metal-dependent hydrolase	stable[3]	
44		3	ABBCDDDDDddEE_______HH_H__HH_H	Type II restriction endonuclease and methyltransferase	core[3]	II
45	8	3	_______D_.____GGGG____________	Type II restriction endonuclease and methyltransferase	intermediate[2], mobile[1]	II
46		3	_BB__D____D____g__G______HH__H	Type II restriction endonuclease and methyltransferase	stable[3]	II
47		3	____D____D_________G__________	incl. alginate O-acetylation protein AlgI	stable[3]	
48		3	ABBC_DDDDDDEEFGGGGGGHHHH_HHHHH	Type III restriction endonuclease and methyltransferase	core[3]	III
49		3	A_____________________________	SAfrica7 specific, incl. Multidrug resistance protein	unique[3]	
50		3	__B_D___D____F_______HH_HHH_HH	incl. P-loop containing NTPase	stable[3]	
51		3	__________________G___________	Cuz20 specific, incl. thiamine pyrophosphokinase	unique[3]	
52		3	ABBCDDDDDDDEEFGGGGGG_HHHHHHH_H	Molybdenum cofactor, Molybdopterin-guanine dinucleotide	core[3]	
53		3	___C__________________________	SJM specific, restriction endonuclease, methyltransferase, Addiction module antidote protein	unique[3]	II
54	1	3	__________________________H___	51 specific; Type IV secretion system, methyltransferase	unique[3]	
55	5	3	ABBCDDDDDDDEEFGGGGGGHHHHHH_HHH	FtsK/SpoIIIE family, nuclease of HNH/ENDO VII superfamily	core[3]	
56	1	3	___________E__________________	India7 specific; Chromosome partitioning protein, cag1	unique[3]	
57	1	3	___________________________H__	F32 specific; Type IV secretion system	unique[3]	
58		3	ABB__DDDDDDEEFGG_G__HHH__HHHHH	Uncharacterized conserved proteins DUF262, DUF1524	core[3]	
59		3	_____________F__________H_____	Methyltransferase	stable[3]	II
60	8	3	_______D______________________	G27 specific; Methyltransferase, glycosyltransferase	unique[3]	III

^a^ Summarization of the occurrence patterns of OGs included in the CGC. Each letter indicates presence/absence of OGs in the CGC in each strain. An upper case letter indicates the strain contains all OGs, a lower case letter indicates the strain contains at least half of the OGs; a period indicates the strain contains less than half of the OGs; an underscore indicates the strains does not contain any OG in the CGC. The strains are ordered in the same way as in S1 Table and Fig 3B. Each strain is indicated in an alphabet according to the phylogeographical group as follows: A, Africa2; B, Africa1; C, SJIM180; D, Europe; E, Asia2; F, PeCan4; G, Amerind; H, EastAsia.

^b^ Mobility classes of OGs in each CGC. The number of OGs in each class is indicated in the brackets.

^c^ Types of RM genes included in each CGC, which are assigned according to the REBASE.

**Table 2:** Neighboring co-occurring gene clusters (NCGCs).

NCGCID	Num OGs	Component CGCs	Comments
1	80	4,6,7,8,12,14,15,19,23,26,33,54,56,57	ICE/TnPZ
2	30	2,5	Bacteriophage
3	25	3,11,24	TerY-P triad cluster
4	12	17,29,42	Cell surface + RMs
5	11	20,35,55	WXG100 secretion system
6	9	16,27	Cluster of P-loop containing NTPases
7	7	28,36	Type II and III RMs
8	6	45,60	Type II and III RMs

The top 5 largest CGCs are also shown in Fig 5B–5F (see S1 Fig for an enlarged version). Here, we assigned an identifier to each CGC based on their size (the number of member OGs), e.g., CGC-1 and CGC-2 for the largest and the second largest CGC, respectively. Similarly, the identifiers of NCGCs are also assigned according to size (the number of member OGs). The largest CGCs and some other characteristic CGCs are described in detail in the next subsection.

We found that 62.0% of Mobile class genes are included in a CGC compared to 42,3% of Intermediate and 32,7% of Core class genes (S2 Fig) suggesting that Mobile class genes tend to cluster on the chromosome more than those of the other classes. Indeed, co-occurrence and clustering of CGCs are typical features of mobile elements. In most cases, genes constituting a CGC are assigned the same mobility class with only few exceptions (Table 1). Thus, we could safely assign a mobility class to each CGC.

In the above phylogenetic network analysis, we identified some unusual clusters that consist of strains in different phylogeographical groups in the Intermediate and Mobile phylogenetic networks (Fig 4E and 4F). Such unusual clusters (marked with a-d in Fig 4E–4F) can now be explained by some of these large CGCs. The cluster a in the Intermediate network (Fig 4E) corresponds to the strains that contain CGC-3 (protein kinase and phosphatase homologs) whereas cluster b corresponds to strains that contain CGC-2 (bacteriophage 1961P). In the network of Mobile class (Fig 4F), both clusters c and d correspond to the patterns related to the integrative conjugative elements (see below), but the situation is rather complex: cluster c corresponds to the strains that do not contain CGC-7, whereas cluster d corresponds to the strains that contain CGC-4 but do not contain CGC-8.

### Some characteristic CGCs

#### *cag* pathogenicity island

The largest CGC (CGC-1) corresponds to the *cag* pathogenicity island (*cag*PAI) that consists of 23 OGs (Fig 5B). This genomic island is absent from two strains, B38 and SouthAfrica7, and is partially deleted in Sat464. Despite some deletion and modification, this cluster is primarily well conserved syntenically and thus classified in Core class. This is consistent with the scenario that the *cag*PAI was once acquired by ancestral *H*. *pylori* and has been inherited through vertical transfer as supported by phylogenetic analysis of *cag*PAI genes [40].

#### Bacteriophage

**Fig 6:**
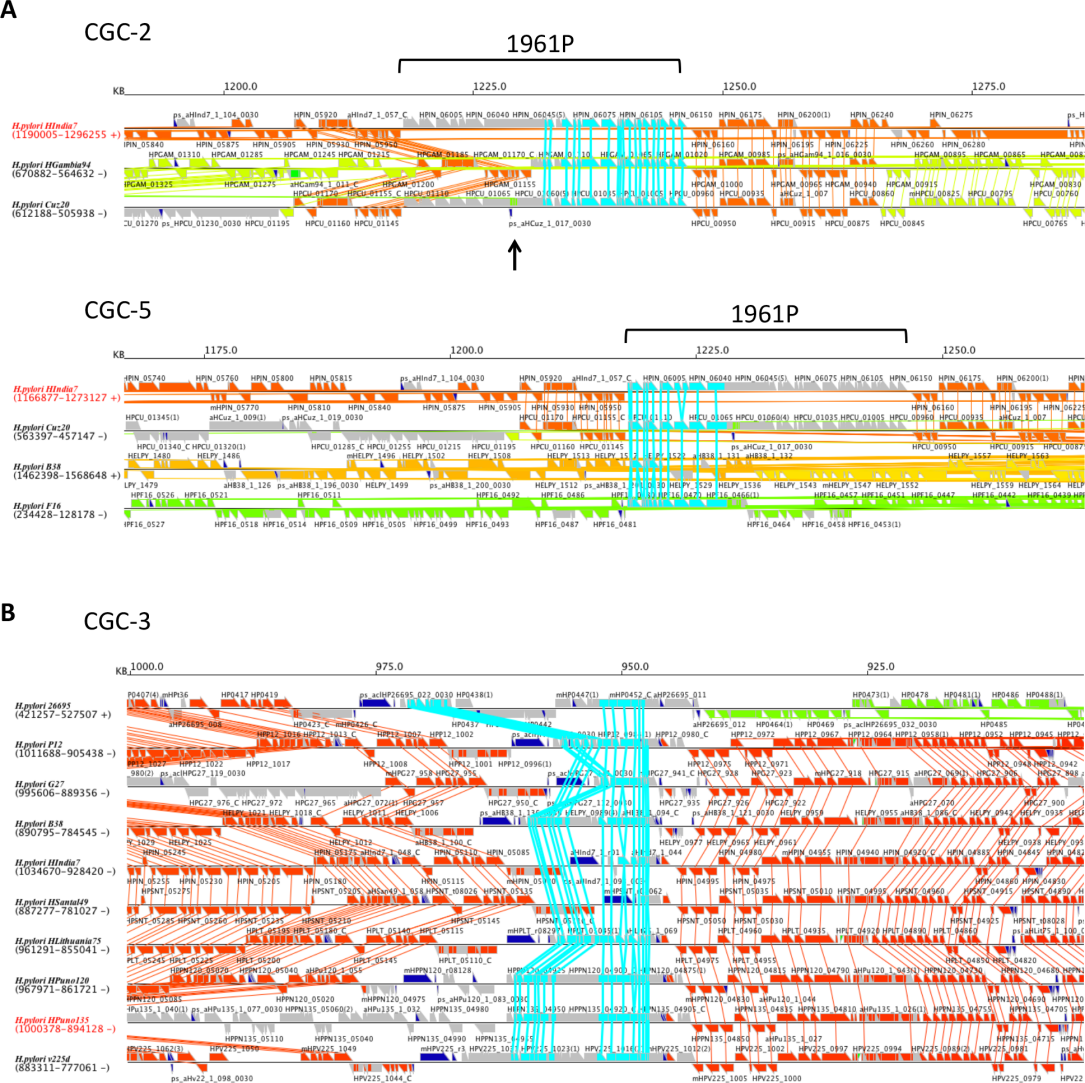
Chromosomal context of CGCs. (A) CGC-2 and CGC-5 (bacteriophage 1961P). (B) CGC-3 (the cluster containing TerY-P triad). Genes in the target CGCs are centered and colored cyan. In the flanking regions, genes in the syntenic core are colored according to the location in the reference genome (whose strain name shown in the left side is colored red). Thus, for a Mobile class CGC (such as CGC-5), the flanking core genes are assigned different colors in different strains.

CGC-2 (19 OGs) and CGC-5 (15 OGs) constitute NCGC-2 and are parts of prophages [41–43] (Fig 5C and 5F). On this phage genome, all ORFs have the same direction with CGC-2 and CGC-5 corresponding to the 3′ half and 5′ half, respectively. As previously reported, only strains Cuz20 and India7 have an apparently complete phage genome whereas F16 and B38 have only the 5′ part and Gambia94/24 contains only the 3′ part (Fig 6A) [41]. Although the copies of this phage are integrated into the same position in Cuz20, India7 and Gambia94/24, CGC-2 is categorized into the Intermediate class because of small segments that are translocated within the phage in Cuz20 (Fig 6A upper). On the other hand, integration sites are different in B38 and F16 and thus CGC-5 is categorized into the Mobile class (Fig 6A lower).

#### An island with eukaryote-type protein kinase/phosphatase and type VII secretion genes

CGC-3 contains a Ser/Thr protein kinase (STK) homolog and a protein phosphatase 2C (PP2C) homolog (Fig 5D). Homologs of these eukaryotic-type protein kinases and phosphatases are found in many bacterial genomes determined so far and are considered to have various physiological roles as components of signaling pathways [44]. This CGC is therefore particularly interesting, in terms of the modulation of protein function through protein phosphorylation/dephosphorylation. In fact, phosphoproteome analysis identified a considerable number of phosphorylation sites on Ser/Thr/Tyr residues within various proteins, including a major virulence factor, vacuolating cytotoxin VacA, in cells of the strain 26695, which contains this CGC [45]. Since there is no other homolog of known STK, this kinase is a candidate factor involved in this phosphorylation process.

**Fig 7:**
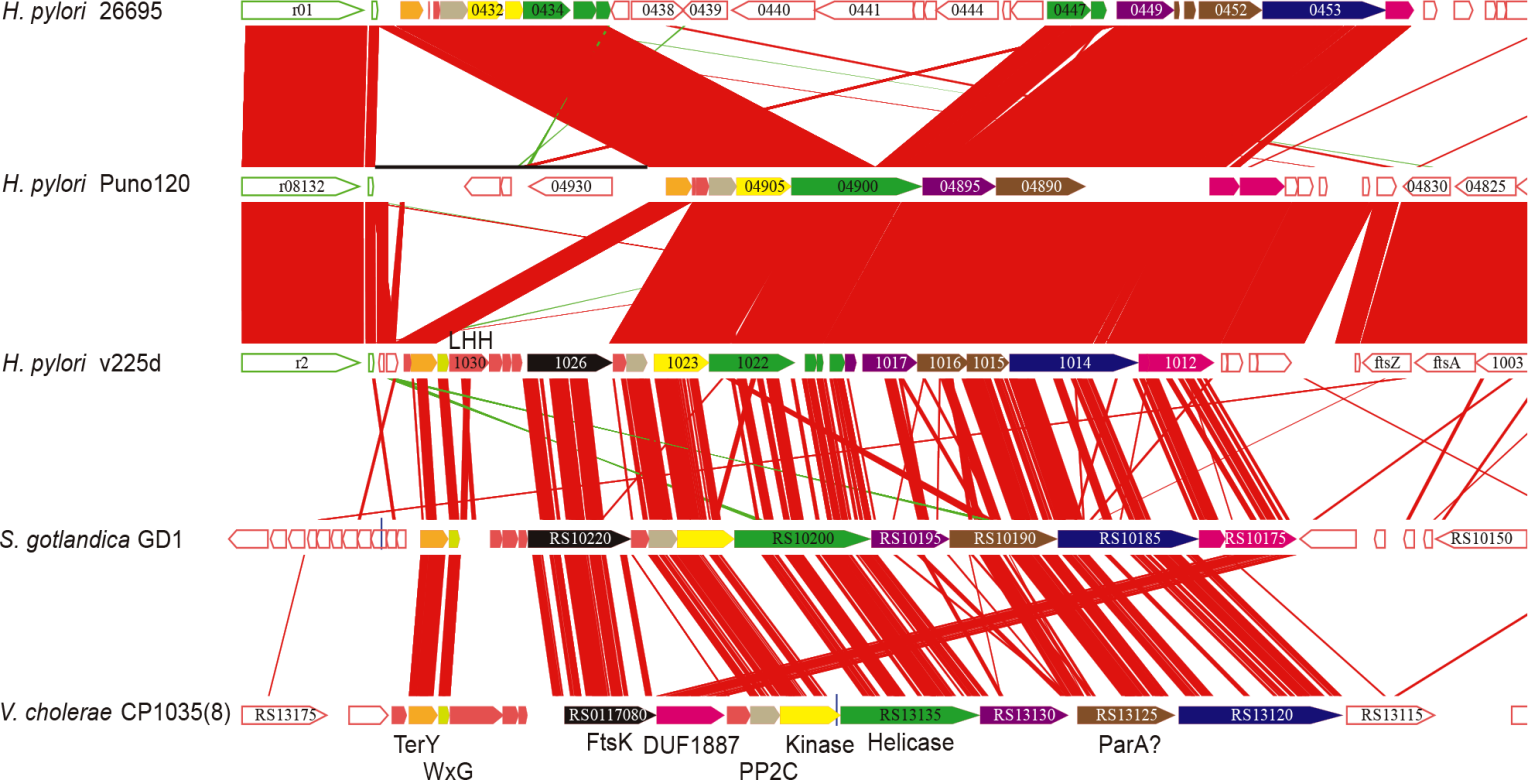
Gene cluster containing TerY-P triad conserved among three *H*. *pylori* strains and two other bacteria. Orthologous genes are drawn with the same colors. Gene numbers or names are presented in or near the arrows. Regions of sequence similarity between loci are indicated by red bands. The diagram was drawn using GenomeMatcher [47].

CGC-3 combined with CGC-11 and CGC-24 constitute NCGC-3, which corresponds to the cluster reported as TerY-phosphorylation (TerY-P) triad [46]. TerY-P triad is composed by three genes: TerY, STY Kinase, and PP2C. Comparison with the cluster found in *Sulfurimonas gotlandica* GD1 (Epsilonproteobacteria) and in *Vibrio cholerae* CP1035(8) (Gammaproteobacteria) revealed that none of the clusters found in *H*. *pylori* contains a complete gene set because of internal deletion and gene disruption (Fig 7). Yet, relatively conserved gene sets are seen in some hspAmerind strains like v225d and Puno120. The reconstructed ancestral cluster encodes WxG, FtsK, AAA+ helicase, LHH nuclease, ParA-like protein (SMC plus McrB), MCRC-NTD (DUF2357) and some uncharacterized proteins besides TerY-P triad. The WxG and FtsK genes, constituting a minimal set of type VII secretion system [46], associated with TerY-P triad are found only in the strain v225d although their paralogs are widely conserved in *H*. *pylori* (e.g., HP0062 and HP0066 in the strain 26695). CGC-3 is categorized into Intermediate class because there is an inversion around this cluster while the other core alignment positions are conserved (Fig 6B).

TerY-P triad and associated DNA-processing modules including DNA-binding proteins, helicases, and some endonucleases are involved in restriction or suicidal action in response to phages and possibly in repairing xenobiotic-induced DNA damage [46]. The TerY-P cluster found in *H*. *pylori* also contains these DNA-binding proteins and enzymes suggesting that the original cluster encoded stress response machinery. The absence of integrase or recombinase in the cluster as well as its stable location in *H*. *pylori* genomes imply that it has lost its mobility.

#### Transposon TnPZ/integrating conjugative element (ICE)

Genomic islands that are integrated in different loci in different strains were previously identified in the plasticity zones of *Helicobacter pylori* genomes, called TnPZs, which contain a cluster of type IV secretion system genes [38, 48]. Distribution of these transposable elements among diverse strains was recently described as integrating conjugative elements (ICE) [49]. The largest NCGC (NCGC-1) consisting of 14 CGCs including CGCs 4, 6, 7 and 8 (Table 2) corresponds to this mobile element. This NCGC contains 9 out of 10 CGCs categorized in Mobile class (Table 1). Typically, these genes are located at one or two positions in each chromosome (S3 Fig).

**Fig 8:**
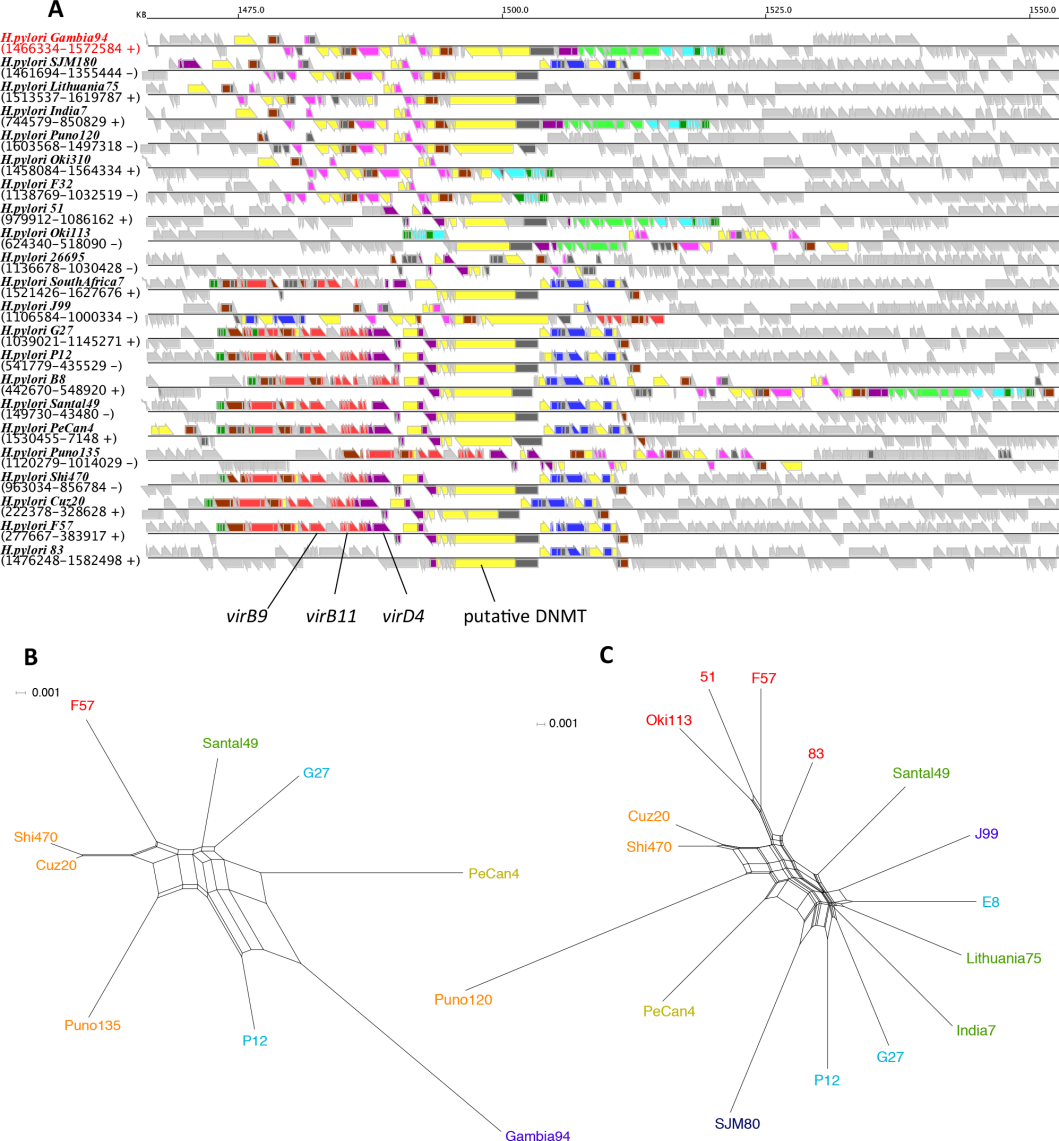
Integrating conjugative elements (ICEs). (A) Locations of ICE genes displayed on the RECOG system. Colors are assigned by CGC groups (CGC-4, red; 6, light green; 7, yellow; 8, magenta; 12, cyan; 15, purple; 19, brown; 23, blue; 26, dark green; other mobile OGs, dark gray). The strains are ordered such that the first 10 strains correspond to type ICE*Hptfs3* and the rest correspond to ICE*Hptfs4*.(B) A phylogenetic network created from the concatenated sequence of three OGs, *virB9*, *virB11* and *virD4*. (C) A phylogenetic network created from the putative DNA methyltransferase (DNMT) conserved in all ICE subtypes. Strain names are assigned colors according to the phylogeographical groups as in Fig 4.

Fig 8A illustrates the gene arrangement of each element, where Mobile class genes are colored according to the CGCs. Previously, two distinct types, designated as ICE*Hptfs3* and ICE*Hptfs4*, with the latter having three subtypes designated as ICE*Hptfs4abc*, were identified [49]. These types can be seen in Fig 8A with prototypical examples in Gambia94/24 (ICE*Hptfs3*) and SouthAfrica7 (ICE*Hptfs4*) (differences among the subtypes of ICE*Hptfs4* are not clearly seen). CGCs specific for ICE*Hpfs3* include CGC-6, CGC-8 and CGC-12 whereas CGCs specific for ICE*Hpfs4* include CGC-4 and CGC-23. CGC-7 is common to both ICE types. In addition, several variants were observed in SJM180, J99 and Puno135 where parts of the two different ICE types are fused. Thus, our approach is useful in identifying and visualizing a family of mobile elements with several structural variants due to its complex evolutionary history. On the basis of the ICE typing, cluster d can be defined by strains containing only ICE*Hpfs4* whereas cluster c can be described as strains containing no ICE.

We constructed phylogenetic networks from the concatenated sequence of *virB9*, *virB11* and *virD4* genes common to ICE*Hptfs4ab* subtypes (Fig 8B) and a putative DNA methylase gene common to all ICE types (Fig 8C). These sequences were previously used to construct phylogenetic trees resulting in trees of similar topologies with an MLST-gene based trees [49]. Accordingly, despite the high mobility of ICE, the overall topologies of these networks are not very different from the topology of the core gene network in that the genes from different phylogeographical groups are generally separated (Fig 8B and 8C). This observation suggests that ICEs have been mainly transferred within the same phylogeographical group.

#### A reverse transcriptase homolog containing cluster

CGC-10 is another cluster that may be interesting (Table 1). It is conserved in 7 to 10 strains and consists of 6 OGs among which four seem to result from one gene split due to gene disruption in some strains. No significant annotation was assigned to these OGs except one that is annotated as phage-associated proteins (COG3600). However, BLAST analysis revealed that the gene spanning the above-mentioned 4 OGs encodes homologs of reverse transcriptase (RT). Several RT homologs were identified in various bacterial genomes and were previously classified into three characterized classes (retrons, group II introns and diversity-generating retroelements) and additional uncharacterized classes [50, 51]. By adding the *H*. *pylori* RT sequence to the phylogenetic analysis of these RT homologs, we found that it is related to retroelements involved in abortive phage infection (Abi), an altruistic suicide of phage-infected cells to prevent secondary infection, which includes *abiA* and *abiK* genes in *Lactococcus* [52] (S4A Fig). AbiK was recently demonstrated to have untemplated DNA polymerase activity that is needed for phage resistance [53].

CGC-10 is identified in less than half of the strains but it spans multiple phylogeographical groups including Europe, EastAsia and Amerind. Since genes in this CGC are categorized into Stable class, they are likely to be inherited from the common ancestor and then lost from the majority of strains. In fact, genes in this cluster are disrupted in 7 out of the 10 strains. In addition, in a phylogenetic network created from the nucleotide sequences of the region conserved among these 10 strains, we can identify clusters corresponding to Europe, EastAsia and Amerind groups (S4B Fig).

#### Restriction-modification genes

Genomes of *H*. *pylori* strains contain diverse sets of restriction-modification (RM) genes [25, 26, 54], each of which has distinct sequence specificity [55]. Many of the RM genes are strain specific and are suggested to be acquired through HGT [56, 57]. RM genes were found as parts of 17 out of 60 CGCs (Table 1). To conduct a systematic survey, we collected 1846 RM genes from REBASE [58] which were classified into 169 OGs with our classification system (S5 Fig). Among them, 18 OGs are conserved in all strains (universal core). Mobility class analyses of the remaining 151 RM OGs revealed that most of them were classified in Core (56 OGs) and Stable (38 OGs) classes, whereas few were in Mobile (4 OGs) and Unique (18 OGs) classes. Thus, despite their diversity, the majority of RM OGs are conserved in multiple strains and located at the equivalent orthologous positions. On the other hand, the remaining 35 OGs are Intermediate class, which can be located in regions flanking the rearrangement boundary or possibly moved to a different locus. Among them are type IV RM genes that are included in CGC-3, part of the TerY-P cluster containing STK and PP2C.

**Fig 9:**
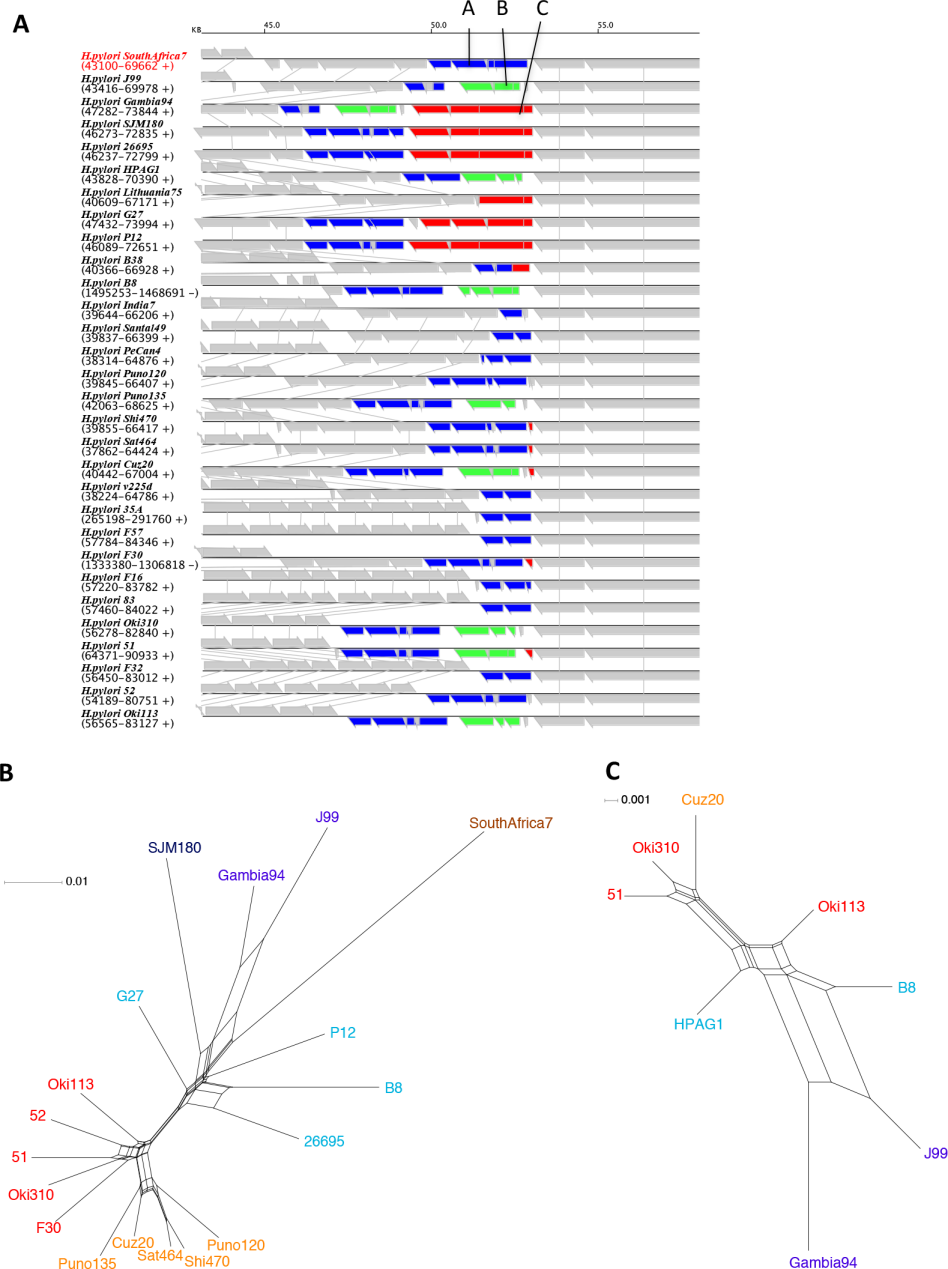
Example of different RM systems occupying the same orthologous position. (A) Location of three RM systems designated A (blue; OG-81, 1424, 1544 and 1524 containing HP0050, HP0051, and HP0052 in strain 26695), B (green; OG-1668, 1667 and 1691 containing jhp0045 and jhp0046 in strain J99), and C (red; OG-1782, 1615, 1727 and 1785 containing HP0053 and HP0054 in strain 26695). See S2 Table for details of each OG. (B) A phylogenetic network created from the concatenated sequence of the RM-A. (C) A phylogenetic network created from the concatenated sequence of the RM-B. Strain names are assigned colors according to the phylogeographical groups as in Fig 4.

Sometimes two stable RM OGs that are conserved in different sets of strains occupy the same orthologous positions. The example illustrated in Fig 9A shows three RM systems: one is core (A) and two are stable (B and C). C is mainly distributed among European strains whereas B is distributed among diverse strains. B and C appear to be mutually exclusive at first glance, but Gambia94/24 strain contains both B and C. Two EastAsia and three Amerind strains also contain short truncated 5´ segments of C in addition to B (Fig 9A). Thus, it is possible that this region once had an ancestral form of C-B-A and subsequently B and/or C were deleted in each strain.

Phylogenetic networks of orthologous groups A and B revealed that whereas the network of A has a very close structure to the core gene network (Fig 9B), the network of B is less similar containing an apparent incongruence that Oki113 is included in the European side rather than the EastAsia/Amerind side (Fig 9C). The cluster B (containing jhp045 and jhp046 in J99 strain) is known to be flanked by a direct repeat [56] which may possibly be involved in an insertion of this RM system into A cluster in a site-specific manner with long target duplication.

## Discussion

Comparison of the core genome sequences can provide valuable information to elucidate evolutionary relationships within a species, not only for tree-like “clonal” evolution but also for non-tree-like evolution, due to mutual homologous recombination [28]. On the other hand, despite its importance in bacterial species diversity, systematic characterization of accessory genome is not easy because of its great diversity. In particular, phylogenetic analysis based on gene content is affected by HGTs, which complicate the interpretation. One previous approach to overcome this problem is to assign a weight to each OG according to the extent of conservation, i.e., the number of genomes containing it [39], to reduce the effect of minority OGs that may have experienced HGTs.

In this work, we developed a simple scheme that consists of mobility class assignment and co-occurring gene clustering to analyze the gene repertoire of a given species, and applied it to characterize the pan-genome among 30 strains of *H*. *pylori*. Our mobility class assignment successfully identified known mobile genes including ICE, prophages and insertion sequences. After eliminating the Mobile class genes and the questionable Intermediate class genes, the phylogenetic network analysis of the remaining Stable and Core class genes showed clustering of the strains in the same phylogeographical groups, consistent with that of the network from the concatenated core sequences (Fig 4), indicating that they have phylogenetic information. Note that many of the minor OGs (conserved only in <50% strains) are Stable and some of the major OGs are Mobile (Fig 3). Thus, our method can better handle mobile genes than that based only on the extent of conservation. Besides the *H*. *pylori* pan genome, this method is expected to be applicable to any other species. Enhancing the generality and usability of the method should be an important future work.

In *H*. *pylori*, the great majority of the genes that are classified as Mobile are clustered in one or two locations in each chromosome that form ICEs. Phylogenetic networks created from the sequences of conserved genes in ICEs retained phylogeographical clusters similar to the core gene network (Fig 8), suggesting that ICEs are rarely transferred between different phylogeographical groups. Nonetheless, ICE’s ability to transfer mainly within the same phylogeographical groups probably enabled the formation of the anomalous phylogenetic patterns observed in various ICE genes. In any case, considering the high homologous recombination rate among *H*. *pylori* genome, it is not easy to elucidate the precise evolutionary history of individual genes that have experienced HGT only on the basis of the conventional phylogenetic analysis.

Traditionally, there are two major approaches to detect HGTs: phylogenetic tree incongruence and anomalous nucleotide composition [59]. Although phylogenetic incongruence can provide strong evidence of HGT, its applicability is limited because it requires a sufficient number of homologs including those closely related to the donor. Instead, many of the previous methods for systematic detection of genomic islands rely on anomalous sequence composition [60, 61]. However, this signal can be weakened after introgression by the amelioration process [62] and can be affected by other factors such as gene expression levels [63]. Both of these methods may not work well for detecting HGTs between the same or very closely related organisms [64]. As an alternative approach, extracting non-conserved regions among closely related genomes, or non-core genes in our terminology, can also be used to identify genomic islands [65, 66] although this approach alone may yield many false positive predictions. To improve the accuracy, combining with additional evidence is effective. For example, known genomic features associated with typical genomic islands, such as tRNA and tmRNA genes, at integration sites was used to extract genomic islands precisely [67, 68]. The mobility class assignment combining with co-occurring gene clustering presented in this work can provide additional evidence for this purpose. Note that a gene being Stable does not mean that it was not acquired by HGT. In fact, it can be once acquired by HGT from other species in some ancestral strain and then be inherited vertically. On the other hand, there is also a possibility that a gene was inserted at a fixed position via a site-specific recombination mechanism, which cannot be detected by our FindMobile procedure. Thus, the mobility analysis presented here provides different information from the conventional methods to detect HGTs. Combining our method with other lines of evidence can promote an understanding of the pan-genome-wide evolution in a given species.

In this work, we classified non-unique and non-core genes into three classes: Stable, Intermediate, and Mobile. Here, we introduced Intermediate class for handling uncertain cases due to rearrangement. We classify an OG into the Intermediate class if it contains a gene located in regions flanking a break point of genome rearrangement, because the genomic context needed to define its mobility extent is broken. Moreover, we also classify OGs of low mobility extent into the Intermediate class because such a case can arise after multiple rearrangement events. In fact, a gene that appears to be transposed can also be explained by double inversions. Thus, genes in Intermediate class are questionable cases in terms of mobility. Nonetheless, some CGCs showing interesting occurrence patterns are classified in the Intermediate class, suggesting that genomic regions flanking the rearrangement boundaries often have high variability and possibly related to mobile gene insertion. Indeed, insertions of mobile genetic elements can promote adjacent genome rearrangements [69]. Further characterization of these genes may be an interesting future project.

## Supporting Information

S1 Fig The five largest CGCs displayed on the RECOG system (enlarged version of Fig 5B–5F). (PDF)

S2 Fig The number of clustered OGs and the total number of OGs in each mobility class. (PDF)

S3 Fig Locations of CGCs 4, 6, 7, 8, 12, 15, 19 23 and 26, corresponding to ICE, on each genome. (PDF)

S4 Fig (A) Phylogenetic tree of reverse transcriptase homologs including the HPSJM_07740 gene of *H*. *pylori* SJM180. (B) Phylogenetic network based on the alignment of nucleotide sequences of CGC-10 genes.
(PDF)

S5 Fig Ortholog table of 169 OGs containing restriction-modification genes displayed in the RECOG system. (PDF)

S1 Table *H*. *pylori* strains used in this study. (DOCX)

S2 Table *H*. *pylori* accessory orthologous groups (OGs). (XLS)
