# Supplementary material for: A Novel Approach to Helicobacter pylori Pan-Genome Analysis for Identification of Genomic Islands
Source: PLoS One. 2016 Aug 9;11(8):e0159419. doi: 10.1371/journal.pone.0159419 (PMC4978471; doi:10.1371/journal.pone.0159419)
Supplement: S1 Fig — (PDF) [file pone.0159419.s001.pdf]

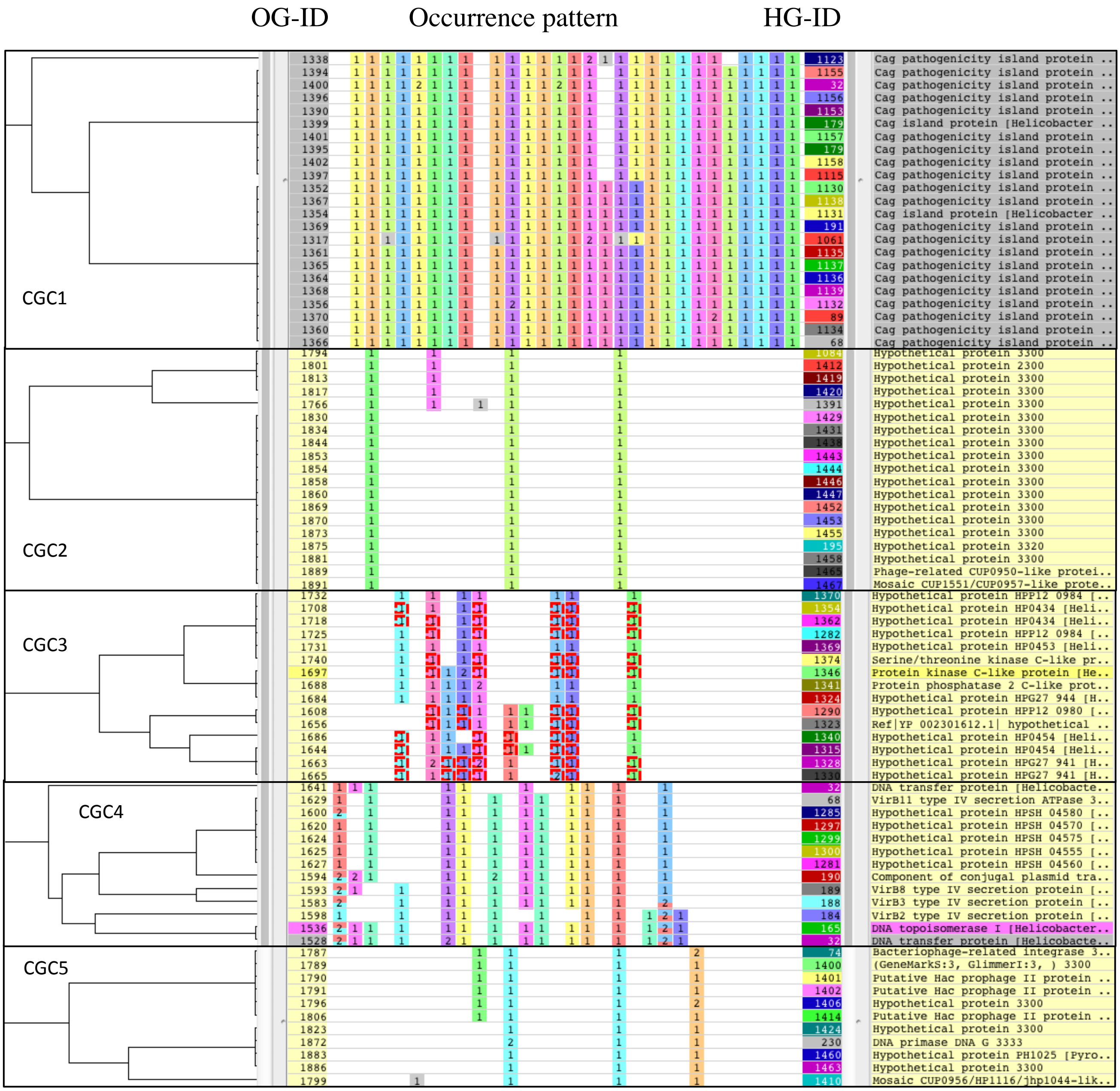

S1 Fig. The five largest CGCs displayed on the RECOG system (enlarged version of Figs 5B-F). Each row represents an ortholog group (OG). Here, the left part contains clustering trees based on occurrence pattern similarity, the central part displays the occurrence pattern table, and the right part displays an annotation of each OG. In the occurrence pattern table, the leftmost column shows ortholog group ID (OG-ID), followed by columns showing the number of in-paralogs in each strains, and the rightmost column shows homolog group ID (HG-ID). Strains are ordered same as in Fig 5 and S1 Table. Here, a color of each cell in the occurrence pattern is assigned on the basis of "Neighboring Clusters" function in RECOG, which assign the same color when the genes located within 10 rows on this table are located within 5000 bases pairs in the same chromosome
