# Supplementary figures and images for: A Novel Approach to Helicobacter pylori Pan-Genome Analysis for Identification of Genomic Islands
Source: PLoS One. 2016 Aug 9;11(8):e0159419. doi: 10.1371/journal.pone.0159419 (PMC4978471; doi:10.1371/journal.pone.0159419)

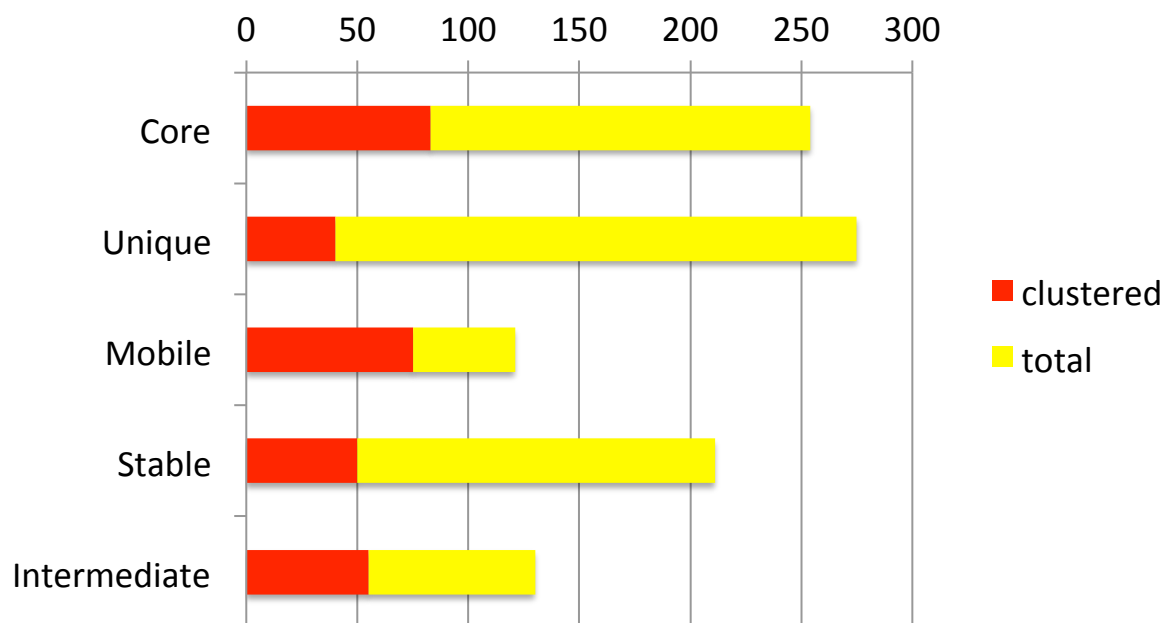

**S2 Fig.** The number of clustered OGs and the total number of OGs in each mobility class.

Supplement: S2 Fig — (PDF) [file pone.0159419.s002.pdf]
