# Supplementary material for: A Novel Approach to Helicobacter pylori Pan-Genome Analysis for Identification of Genomic Islands
Source: PLoS One. 2016 Aug 9;11(8):e0159419. doi: 10.1371/journal.pone.0159419 (PMC4978471; doi:10.1371/journal.pone.0159419)
Supplement: S3 Fig — (PDF) [file pone.0159419.s003.pdf]

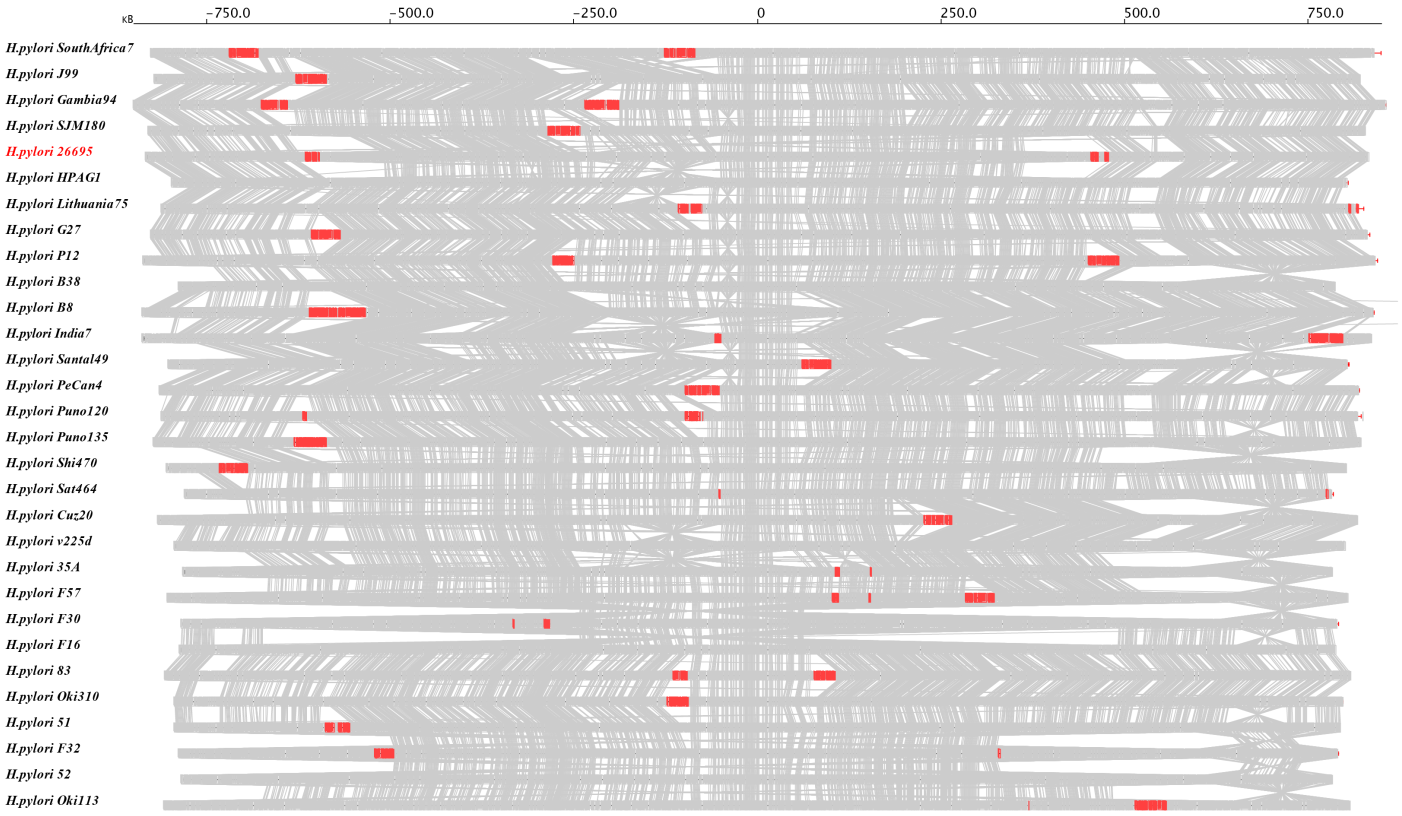

S3 Fig. Locations of CGCs 4, 6, 7, 8, 12, 15, 19 23 and 26, corresponding to ICE, on each genome. Genomes are aligned such that HP0001 (nusB) in strain 26695 and its orthologs (which are defined as the first ORFs in most of the genomes) are located at the center. The coordinate shown is the relative position from the center.
