# Supplementary material for: A Novel Approach to Helicobacter pylori Pan-Genome Analysis for Identification of Genomic Islands
Source: PLoS One. 2016 Aug 9;11(8):e0159419. doi: 10.1371/journal.pone.0159419 (PMC4978471; doi:10.1371/journal.pone.0159419)
Supplement: S4 Fig — (A) Phylogenetic tree of reverse transcriptase homologs including the HPSJM_07740 gene of H. pylori SJM180. (B) Phylogenetic network based on the alignment of nucleotide sequences of CGC-10 genes. (PDF) [file pone.0159419.s004.pdf]

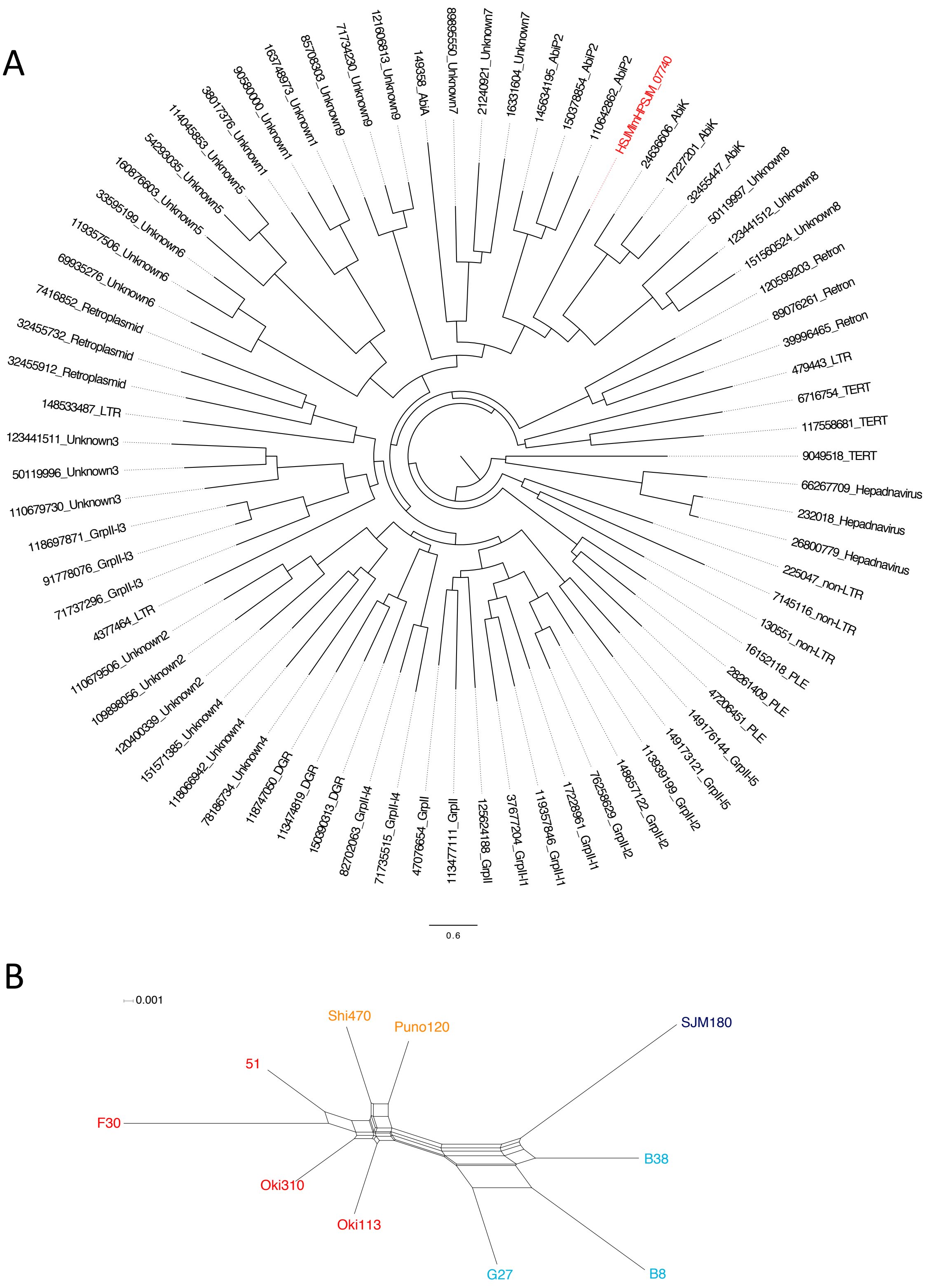

S4 Fig. (A) Phylogenetic tree of reverse transcriptase homologs including the HPSJM_07740 gene of *H. pylori* SJM180. (B) Phylogenetic network based on the alignment of nucleotide sequences of CGC-10 genes. Strain names are assigned colors according to the phylogeographical groups as in Fig 4.
