# Supplementary material for: A Novel Approach to Helicobacter pylori Pan-Genome Analysis for Identification of Genomic Islands
Source: PLoS One. 2016 Aug 9;11(8):e0159419. doi: 10.1371/journal.pone.0159419 (PMC4978471; doi:10.1371/journal.pone.0159419)
Supplement: S5 Fig — (PDF) [file pone.0159419.s005.pdf]

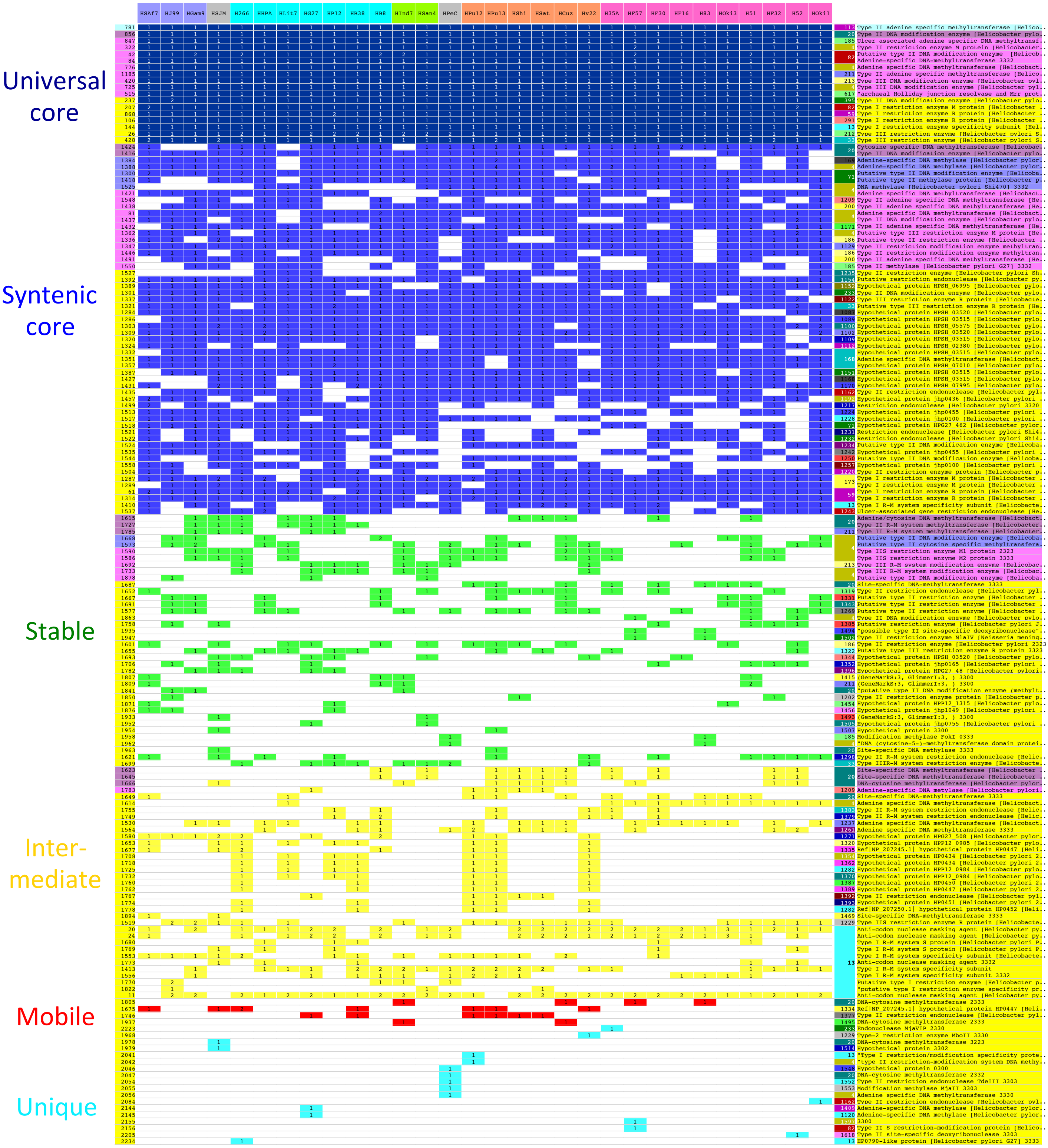

S5 Fig. Ortholog table of 169 OGs containing restriction-modification genes displayed in the RECOG system.
