# Supplementary material for: A Novel Approach to Helicobacter pylori Pan-Genome Analysis for Identification of Genomic Islands
Source: PLoS One. 2016 Aug 9;11(8):e0159419. doi: 10.1371/journal.pone.0159419 (PMC4978471; doi:10.1371/journal.pone.0159419)
Supplement: S1 Table — (DOCX) [file pone.0159419.s006.docx]

**S1** **Table** *H. pylori* strains used in this study

| **strain** | **Accession No.** | **subgroup** |
| --- | --- | --- |
| SouthAfrica7 | CP002336, CP002337 | Africa2_sg1 |
| J99 | NC_000921 | Africa1_sg1 |
| Gambia94 | CP002332, CP002333 |  |
| SJM180 | NC_014560 | singleton (hybrid) |
| 26695 | NC_000915 | Europe_sg1 |
| HPAG1 | NC_008086, NC_008087 |  |
| Lithuania75 | CP002334, CP002335 |  |
| G27 | NC_011333 | Europe_sg2 |
| P12 | NC_011498, NC_011499 |  |
| B38 | NC_012973 |  |
| B8 | NC_014257, NC_014256 |  |
| India7 | CP002331 | Asia2_sg1 |
| Santal49 | CP002983, CP002984 |  |
| PeCan4 | NC_014555, NC_014556 | singleton (hybrid) |
| Puno120 | CP002980, CP002981 | Amerind_sg1 |
| Puno135 | CP002982 |  |
| Shi470 | NC_010698 | Amerind_sg2 |
| Sat464 | CP002071, CP002072 |  |
| Cuz20 | CP002076 | singleton (Amerind_sg3) |
| v225d | CP001582, CP001583 | singleton (Amerind_sg4) |
| 35A | CP002096 | EastAsia_sg1 |
| F57 | AP011945 |  |
| F30 | AP011941, AP011942 |  |
| F16 | AP011940 |  |
| 83 | CP002605 | EastAsia_sg2 |
| OK310 | AP012601, AP012602 |  |
| 51 | CP000012 |  |
| F32 | AP011943, AP011944 | EastAsia_sg3 |
| 52 | CP001680 |  |
| OK113 | AP012600 | singleton (EastAsia_sg4) |
